# A gamified interactive E-book incorporating metacognitive self-regulation: effects on physics achievement, learning motivation, and metacognitive self-regulation ability in junior high school

**DOI:** 10.3389/fpsyg.2026.1832021

**Published:** 2026-05-21

**Authors:** Jiawei Shao, Shupeng Tang, Zhou Zhuang

**Affiliations:** 1 Centre for Instructional Technology and Multimedia, Universiti Sains Malaysia, Penang, Malaysia; 2 School of Educational Studies, Universiti Sains Malaysia, Penang, Malaysia; 3 School of Physical Education, Changzhou University, Changzhou, China

**Keywords:** gamified interactive E-book (GIEB), gamified learning design, learning motivation, metacognitive self-regulation strategies (MSRS), physics academic achievement, self-regulated learning

## Abstract

Although metacognitive self-regulation strategies (MSRS) have demonstrated robust effects on learning outcomes, their implementation in traditional classrooms often yields inconsistent results due to insufficient motivational support and delayed feedback. This study investigated whether gamification can serve as motivational infrastructure to sustain MSRS implementation in junior high school physics education. A Gamified Interactive E-Book (GIEB) was designed, grounding metacognitive scaffolding (planning–monitoring–reflection) within a gamified environment informed by Self-Determination Theory and Zimmerman’s cyclical model of self-regulated learning. Using a quasi-experimental pretest–posttest design, 60 eighth-grade students in China were assigned to either the GIEB group (*n* = 30) or a traditional lecture group receiving oral metacognitive strategy instruction (*n* = 30). ANCOVA results indicated that the GIEB group demonstrated significantly greater improvements in physics achievement (*F* = 29.165, *p* < 0.001, η^2^ = 0.338), learning motivation (*F* = 22.832, *p* < 0.001, η^2^ = 0.286), and metacognitive self-regulation ability (*F* = 19.956, *p* < 0.001, η^2^ = 0.259). The TL group showed no significant gains on any measure. These findings suggest that gamified environments providing structured scaffolding, immediate feedback, and progress visualization may address the motivational bottleneck that limits MSRS effectiveness in conventional instruction. Implications for designing technology-enhanced metacognitive interventions in science education are discussed.

## 1 Introduction

### 1.1 The challenge of physics learning in junior high school

Physics occupies a distinctive position in science education owing to its abstract epistemological framework and strong explanatory power (Maison et al., 2019). Alongside chemistry and biology, it constitutes a core component of science curricula worldwide, providing essential principles for understanding natural phenomena and driving technological innovation (Cho and Baek, 2019). The junior high school years (approximately ages 11–15) represent a critical period for physics education: this developmental stage coincides with the rapid growth of students’ cognitive capacities and the formation of scientific interests and learning habits (OECD, 2019; Mullis et al., 2016; Redish, 2014). Early structured physics instruction at this stage enhances scientific reasoning, critical thinking, and inquiry skills, establishing a foundation for subsequent STEM learning (Syeda and Zahid, 2024; Ofek-Geva and Fortus, 2024).

However, due to the inherently abstract nature of physics and its high cognitive demands—including advanced reasoning, logical analysis, and mathematical modeling—many students encounter considerable difficulties (Maison et al., 2019; Vidak et al., 2024; Shrestha et al., 2023; Zulkiffli et al., 2024). Large-scale international assessments such as PISA and TIMSS consistently confirm this challenge: despite substantial investment in secondary physics education, students’ performance shows significant room for improvement across many countries (OECD, 2019; Mullis et al., 2020; Mullis et al., 2023; OECD, 2023). These persistent learning difficulties may undermine the development of scientific literacy, restrict problem-solving and critical thinking abilities, and hinder future engagement with STEM fields (Maltese and Tai, 2011; Maison et al., 2019). Identifying effective instructional interventions to enhance junior high school students’ physics learning outcomes has therefore become a pressing priority.

### 1.2 Metacognitive self-regulation strategies: established benefits and persistent implementation challenges

Metacognitive self-regulation (MSR) refers specifically to the regulation of cognitive activities during learning, including planning, monitoring, evaluation, and adjustment (Pintrich, 2000). Distinguished from the broader constructs of metacognition (Flavell, 1979) and self-regulated learning (SRL; Zimmerman, 2000), MSR was adopted in the present study for its theoretically precise construct, pedagogical operability, and measurability within strategy-oriented instructional designs (Tock and Moxley, 2017; Dinsmore et al., 2008; Veenman et al., 2006).

The effectiveness of metacognitive self-regulation strategies (MSRS) is well established. Extensive empirical research confirms positive effects across educational stages, disciplines, and cultural contexts, demonstrating benefits for motivation, metacognitive awareness, self-efficacy, and academic achievement. For example, Lavasani et al. (2011) showed that systematic MSRS instruction significantly enhanced motivation and self-efficacy among primary students; Kramarski (2008) reported improvements in metacognitive regulation and problem-solving performance among teachers; and interventions at secondary and tertiary levels have consistently been associated with gains in achievement, autonomy, and strategic learning behaviors (Zhang, 2024; Erlin et al., 2024; Baissane and Zaid, 2024; Katsantonis and McLellan, 2023; Wang and Sperling, 2020). With advances in educational technology, embedding MSRS into digital environments has shown increasing promise, with learning dashboards, collaborative scripts, and AI-supported feedback systems strengthening engagement in planning, monitoring, and reflection (Wang and Sperling, 2020; Chen and Chiu, 2016; Karaoğlan Yılmaz et al., 2018).

Despite these documented benefits, the implementation of MSRS faces a critical and persistent challenge: their effectiveness is highly contingent on the instructional context in which they are delivered. Several studies report null or limited effects, often attributed to insufficient scaffolding, delayed feedback, or abstract strategy instruction disconnected from authentic learning tasks (Henriksson, 2017; Eggers et al., 2021). The Education Endowment Foundation (2020) similarly emphasized that metacognitive instruction yields substantial gains only when accompanied by explicit modeling, timely feedback, and contextualized application. Critically, research has identified a declining trend in MSR during adolescence, particularly in secondary school, a decline that appears closely linked to deteriorating learning motivation (Katsantonis, 2024; Ahmed et al., 2013; Bardach et al., 2023). Katsantonis (2024) argued that declining motivation serves as a critical driver of deteriorating MSR, creating a negative feedback loop: as motivation decreases, students’ willingness and capacity to engage in effortful regulatory behaviors diminishes, which in turn leads to poorer learning outcomes and further motivational decline.

This evidence points to a fundamental bottleneck: the sustained implementation of MSRS requires continuous motivational support and immediate feedback mechanisms that traditional teacher-centered instruction struggles to provide. Within conventional classroom settings, metacognitive strategy instruction is typically delivered through oral explanation or static worksheets, offering limited opportunities for timely feedback, learner autonomy, or visible progress tracking. As a result, students are prone to disengagement, boredom, and reduced intrinsic motivation, all of which undermine the development and application of self-regulatory processes (Mega et al., 2014; Tze et al., 2016; Mann and Robinson, 2009). The question thus becomes: what kind of instructional environment can provide the motivational infrastructure needed to activate and sustain MSRS?

### 1.3 Gamification as motivational infrastructure for metacognitive self-regulation

Gamification—the incorporation of game design elements into non-game contexts (Deterding et al., 2011)—has emerged as a widely adopted motivational strategy in education (Swacha, 2021; Koivisto and Hamari, 2019). A growing body of evidence demonstrates that gamification can enhance students’ academic achievement, learning motivation, engagement, and self-regulation (Sailer and Homner, 2020; Alsawaier, 2018; Chen C. et al., 2024). In physics education specifically, gamification shows particular promise by transforming abstract concepts into interactive learning experiences through simulations and inquiry-based tasks (Richter and Kickmeier-Rust, 2025; Feger et al., 2019; Ab Patar et al., 2024).

Crucially, gamification aligns with the core processes of self-regulated learning. From a mechanistic perspective, gamification mechanisms such as goal setting, immediate feedback, progress visualization, and staged challenges map directly onto the planning, monitoring, and reflection phases of cyclical SRL models (Qiao et al., 2022; Tang and Kay, 2014). Tang and Kay (2014) argued that gamification should function not merely as a motivational device but as a metacognitive scaffold, embedding mechanisms that facilitate strategic planning, real-time monitoring, and reflective evaluation. This perspective positions gamification as a potential solution to the implementation bottleneck identified above: by providing continuous motivational support and immediate, visible feedback, gamified environments may create the conditions necessary for MSRS to be effectively enacted and sustained.

Empirical studies have begun to examine this integration. Chen C. et al. (2024) designed a gamified interactive e-book embedding MSRS in a flipped mathematics classroom for Chinese elementary students and reported significant gains in achievement, motivation, and self-regulation. Al-Hosni et al. (2024) found that gamified mobile applications incorporating badges, points, and competitive modules significantly improved Grade 8 students’ metacognitive regulation. Ateş and Polat (2025) demonstrated that combining augmented reality with MSRS-based gamification led to higher achievement, engagement, and self-efficacy compared to gamification alone. Chen Y. C. et al. (2024) reported significant gains in mathematics achievement, goal setting, and reflection through a gamified mobile MSR intervention, although improvements in monitoring were not observed.

However, findings remain mixed. Ferreira da Rocha et al. (2024) found no significant effects of a gamified intelligent tutoring system on SRL or performance, attributing the null results to overly simplified gamification designs (points-only systems) and short intervention duration. Smith et al. (2023), in a Finnish university context, found that while open gamification prompts enhanced reflective awareness, overly structured feedback in some cases suppressed regulatory outcomes. These inconsistencies suggest that the effectiveness of MSRS-gamification integration depends critically on design quality—specifically, whether gamification elements provide genuine scaffolding for regulatory processes or merely superficial reward mechanisms.

### 1.4 Research gaps and the present study

Despite encouraging preliminary evidence, several significant gaps remain. First, empirical studies that systematically integrate MSRS with gamification in junior high school physics contexts are scarce; most existing work targets elementary education, higher education, or non-physics subjects. Second, many gamified interventions rely on simplified reward systems (e.g., points only) without embedding structured metacognitive scaffolding, limiting their capacity to support deep regulatory engagement. Third, existing research in the Chinese educational context remains limited, despite the large student population and the policy emphasis on strengthening physics education (Ministry of Education of the People’s Republic of China, 2022, 2025).

To address these gaps, the present study designed and evaluated a Gamified Interactive E-Book (GIEB) that integrates metacognitive self-regulation strategies with gamification features for eighth-grade physics education in China. The central aim was to compare two contrasting modes of MSRS implementation: a technology-enhanced mode in which metacognitive scaffolding is embedded within a gamified, interactive digital environment, versus a conventional classroom mode in which the same metacognitive strategies are delivered through oral teacher instruction. Grounded in Zimmerman’s (2000, 2002) cyclical model of self-regulated learning as the primary theoretical framework and informed by Self-Determination Theory (Deci and Ryan, 2000), the GIEB embeds structured planning, monitoring, and reflection scaffolds within a gamified environment that provides autonomy-supportive task design, competence-signaling feedback, and visualized progress tracking.

Based on the theoretical rationale and prior empirical evidence, the following hypotheses were proposed regarding the comparative effectiveness of these two MSRS implementation modes:

H1: Students using the GIEB will demonstrate significantly greater improvement in learning motivation compared to students receiving traditional lecture-based instruction with oral metacognitive strategy guidance.

H2: Students using the GIEB will demonstrate significantly greater improvement in metacognitive self-regulation ability compared to students receiving traditional lecture-based instruction with oral metacognitive strategy guidance.

H3: Students using the GIEB will demonstrate significantly greater improvement in physics achievement compared to students receiving traditional lecture-based instruction with oral metacognitive strategy guidance.

## 2 The GIEB design process

### 2.1 System architecture of GIEB

The Gamified Interactive E-Book (GIEB) employed in this study was developed on the YOYA platform (Xiamen Yoya Network Technology Co., Ltd.), which supports multimedia integration, interactive assessment, and animated instructional design. This platform provides a stable technical foundation for implementing a gamified learning environment that integrates metacognitive self-regulation strategies (MSRS) to support junior high school students’ engagement in physics learning.

**Figure 1:**
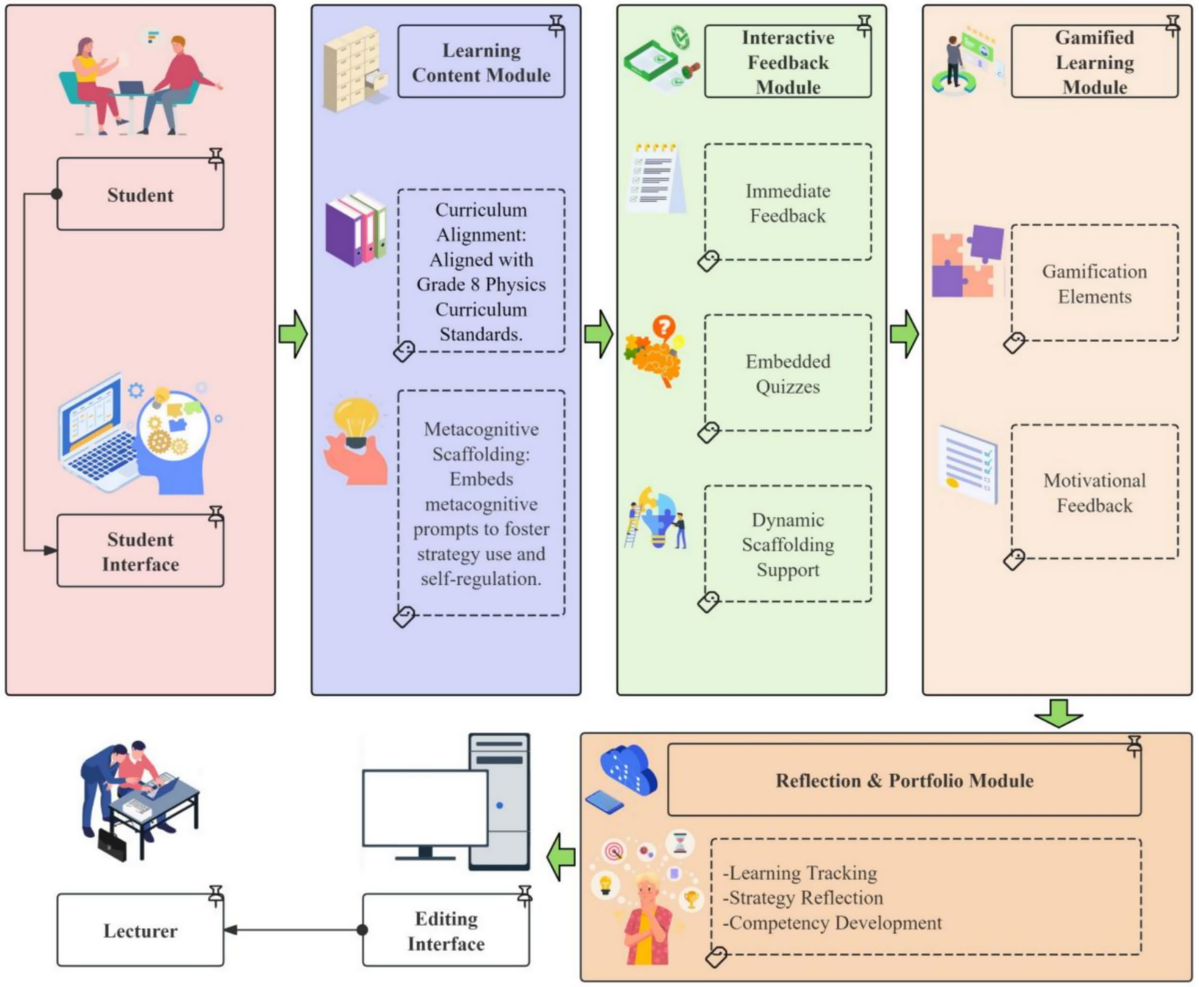
System architecture of GIEB.

The GIEB consists of four core modules—Learning Content Module, Interactive Feedback Module, Gamified Learning Module, and Reflection & Portfolio Module—which together form a multidimensional learning environment combining metacognitive scaffolding with motivational support (see Figure 1).

#### 2.1.1 Learning content module

Aligned with China’s junior high school physics curriculum standards, this module embeds metacognitive prompts corresponding to the stages of planning, monitoring, and evaluation/regulation, guiding students to approach learning tasks strategically and to develop self-regulatory awareness.

#### 2.1.2 Interactive feedback module

This module provides immediate feedback, embedded formative assessments, and adaptive scaffolding to support students’ real-time monitoring of comprehension and strategy use, facilitating ongoing evaluation and adjustment during task engagement.

#### 2.1.3 Gamified learning module

This module incorporates gamification elements such as points, badges, contextualized tasks, and motivational feedback. These elements function as informational cues that signal learning progress, task completion, and strategy use. Consistent with Self-Determination Theory (SDT), the module adopts an autonomy-supportive design by preserving learner choice, providing competence-related feedback, and enhancing the visibility of learning progress, thereby supporting sustained engagement without imposing controlling incentives.

#### 2.1.4 Reflection and portfolio module

This module records students’ learning behaviors, task completion, and trajectories of MSRS use, presenting progress data in a visualized format. It supports students’ reflection on learning processes and strategy adjustment, while also providing teachers with diagnostic information to inform instructional decisions.

Overall, the GIEB architecture integrates metacognitive self-regulation processes with motivationally supportive features, coordinating cognitive, affective, and behavioral dimensions to provide structured support for students’ strategy use and engagement in junior high school physics learning.

### 2.2 Theoretical framework of the GIEB

The Gamified Interactive E-Book (GIEB) developed in this study is grounded in Self-Determination Theory (SDT; Deci and Ryan, 2000) as its primary motivational framework, and further informed by the gamification design framework proposed by Kam and Umar (2018) and the Metacognitive Scaffolding Gamification Framework introduced by Tang and Kay (2014). Together, these perspectives guide the design of a technology-enhanced learning environment that supports junior high school students’ learning motivation, metacognitive self-regulation, and physics achievement.

According to Self-Determination Theory, sustained learning engagement is fostered when learners’ basic psychological needs for autonomy, competence, and relatedness are supported (Deci and Ryan, 2000; Reeve, 2012). Following Kam and Umar’s (2018) framework, the GIEB emphasizes autonomy support through meaningful choices and flexible learning pathways, competence facilitation through appropriately challenging tasks and competence-related feedback, and relatedness development through social visibility and interaction mechanisms embedded in the learning environment.

Beyond motivational support, the GIEB also adopts the Metacognitive Scaffolding Gamification Framework (Tang and Kay, 2014), which conceptualizes gamification as a scaffold for implementing core metacognitive self-regulation processes. In line with this framework, the GIEB integrates goal-setting supports at the beginning of learning units, provides in-task feedback to facilitate self-monitoring, and incorporates reflective prompts to encourage evaluation and strategy adjustment.

Importantly, the integration of motivational and metacognitive mechanisms in the GIEB is explicitly aligned with Zimmerman’s cyclical model of self-regulated learning (Zimmerman, 2000; Zimmerman, 2011). Within this model, self-regulated learning unfolds across three interrelated phases—forethought, performance, and self-reflection. The GIEB operationalizes these phases by supporting strategic planning during forethought, monitoring and strategy execution during performance, and reflective evaluation and adaptive regulation during self-reflection. In this way, gamification functions as a design scaffold that supports engagement across the full SRL cycle rather than as an isolated motivational add-on.

**Figure 2:**
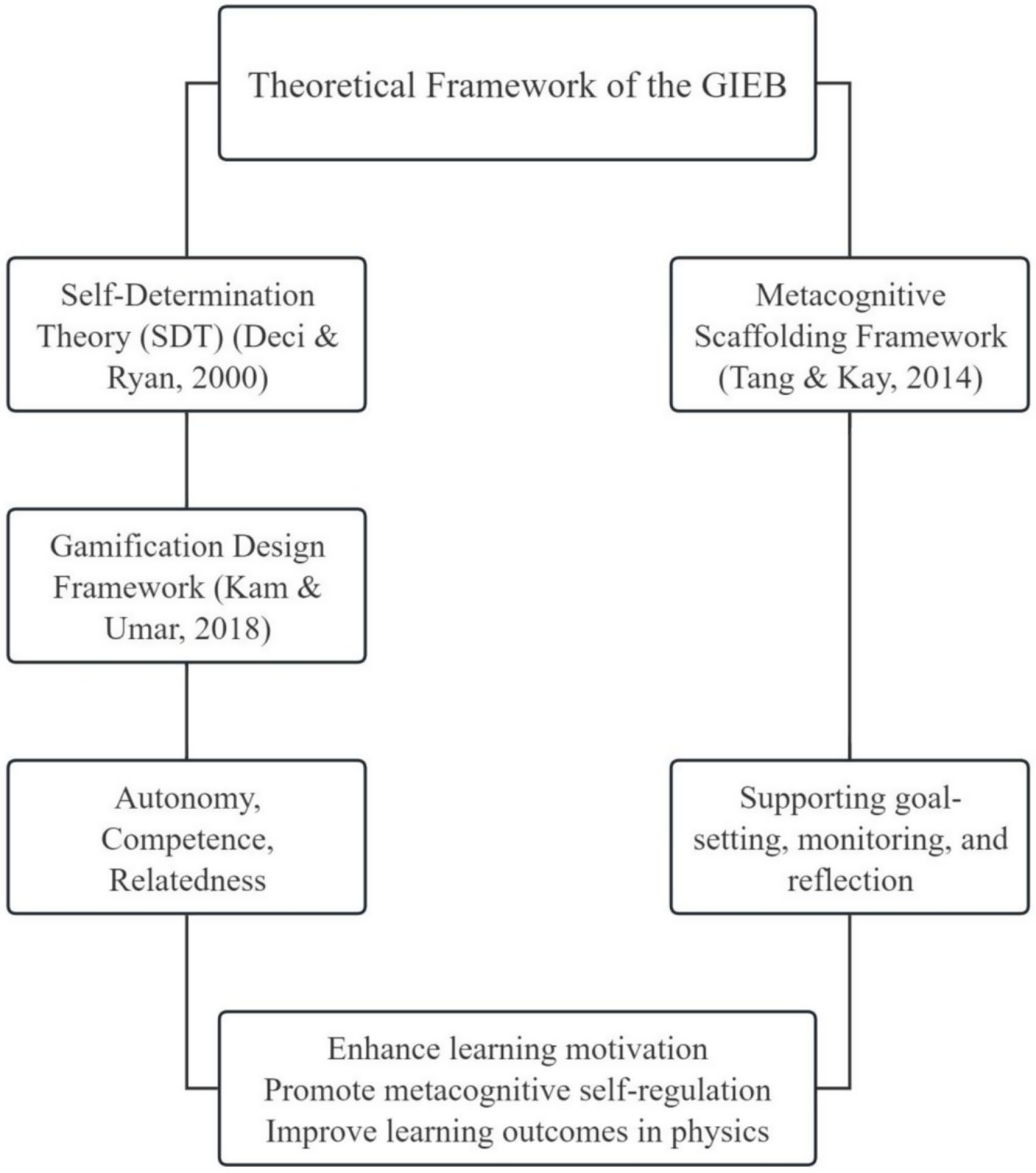
Theoretical integration framework of the gamified interactive e-book (GIEB).

In summary, the GIEB integrates motivational principles from SDT with metacognitive scaffolding strategies grounded in contemporary SRL theory. By explicitly aligning gamification mechanisms with Zimmerman’s cyclical SRL model, the theoretical framework provides a coherent and parsimonious rationale for the system design (see Figure 2).

### 2.3 Development phases of the GIEB

**Figure 3:**
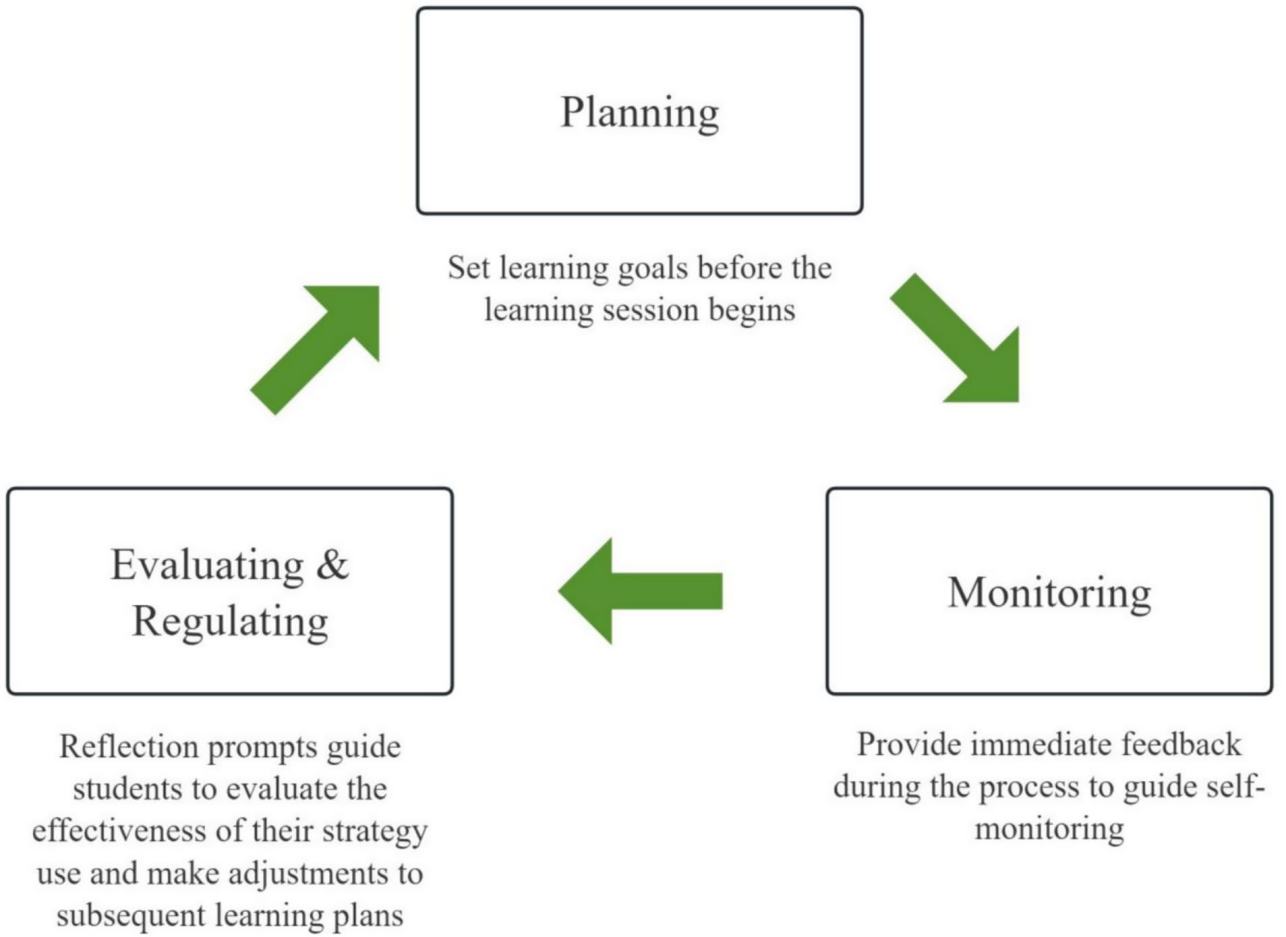
The cyclical self-regulation process embedded in the GIEB learning design.

When students engage with the Gamified Interactive E-Book (GIEB), each learning activity follows a cyclical sequence of self-regulation phases—planning, monitoring, and evaluation/regulation. These phases correspond to the core processes of metacognitive self-regulation and are explicitly aligned with Zimmerman’s cyclical model of self-regulated learning, in which learning unfolds across forethought, performance, and self-reflection phases. Rather than operating linearly, these processes form a recursive cycle in which reflections generated during evaluation inform subsequent goal setting and strategic planning, resulting in a spiral pathway of “planning–monitoring–evaluating–replanning” (see Figure 3).

Throughout this process, the GIEB integrates three functional modules—Learning Content, Interactive Support, and Gamified Learning—to provide coordinated cognitive scaffolding and motivational support. The Learning Content Module offers curriculum-aligned resources and contextualized tasks, embedding metacognitive prompts that guide goal setting, strategy selection, and reflective evaluation. The Interactive Support Module provides immediate feedback, metacognitive prompts, and adaptive scaffolding to facilitate continuous self-monitoring and strategy adjustment during task engagement.

The Gamified Learning Module incorporates goal-oriented mechanisms such as points, achievement displays, and personalized feedback. Consistent with an autonomy-supportive design informed by Self-Determination Theory, these elements function as informational cues that signal learning progress and strategy use rather than as controlling incentives, thereby supporting sustained engagement across self-regulation phases.

In addition, the Learning Log and Portfolio Module records students’ behavioral data, task completion, and trajectories of strategy use in real time. Progress is presented in a visualized format that supports systematic reflection and strategy optimization by students, while also providing teachers with diagnostic information to inform instructional decisions.

**Figure 4:**
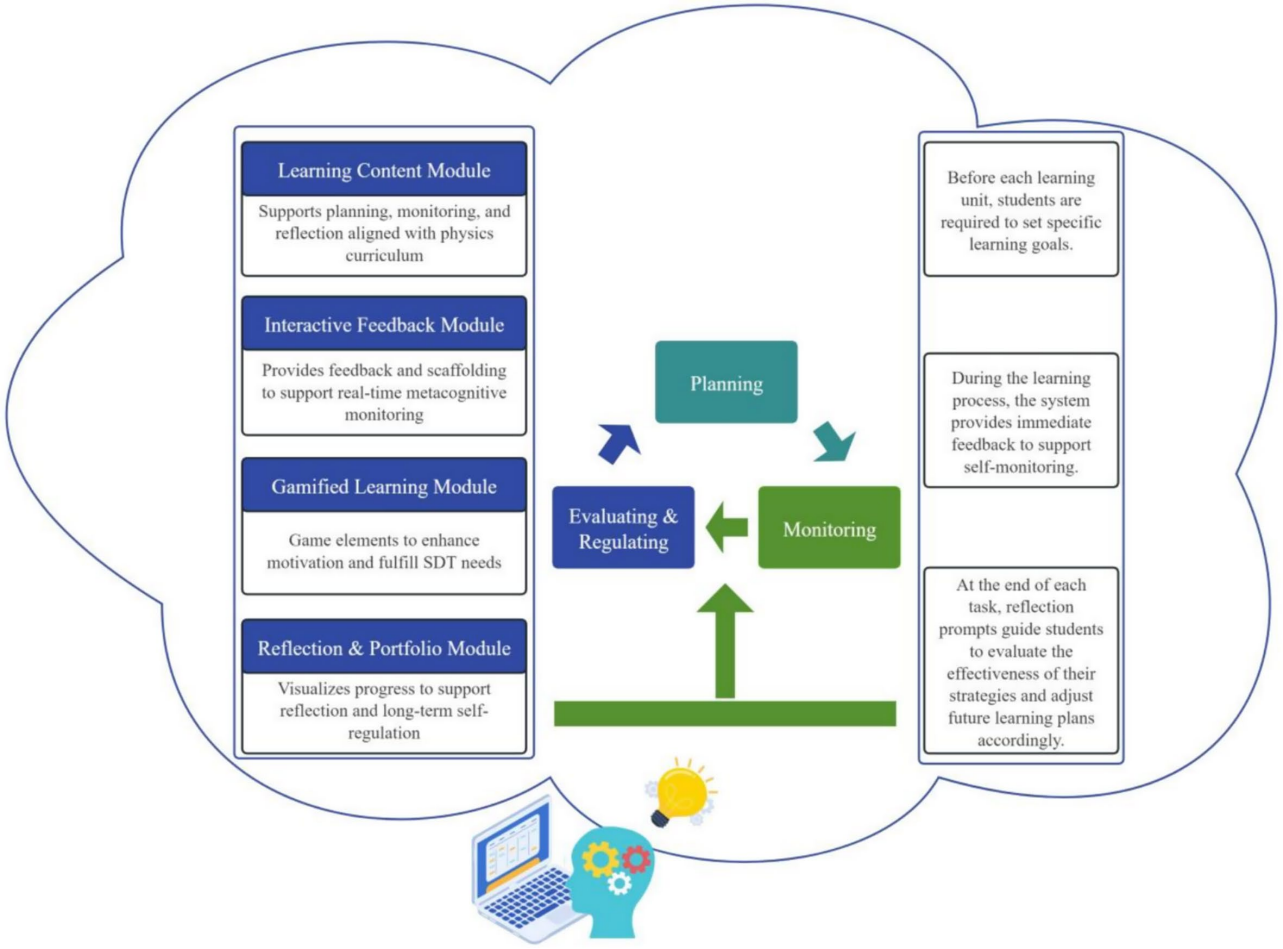
The design model integrating functional modules with cyclical phases of metacognitive self-regulation.

In summary, the GIEB operationalizes metacognitive self-regulation through a structured yet flexible SRL cycle that integrates motivationally supportive features with cognitive scaffolding. This design provides a coherent pathway for supporting students’ regulation of learning in junior high school physics (see Figure 4).

#### 2.3.1 Planning phase: activating goal awareness and cognitive readiness

**Figure 5:**
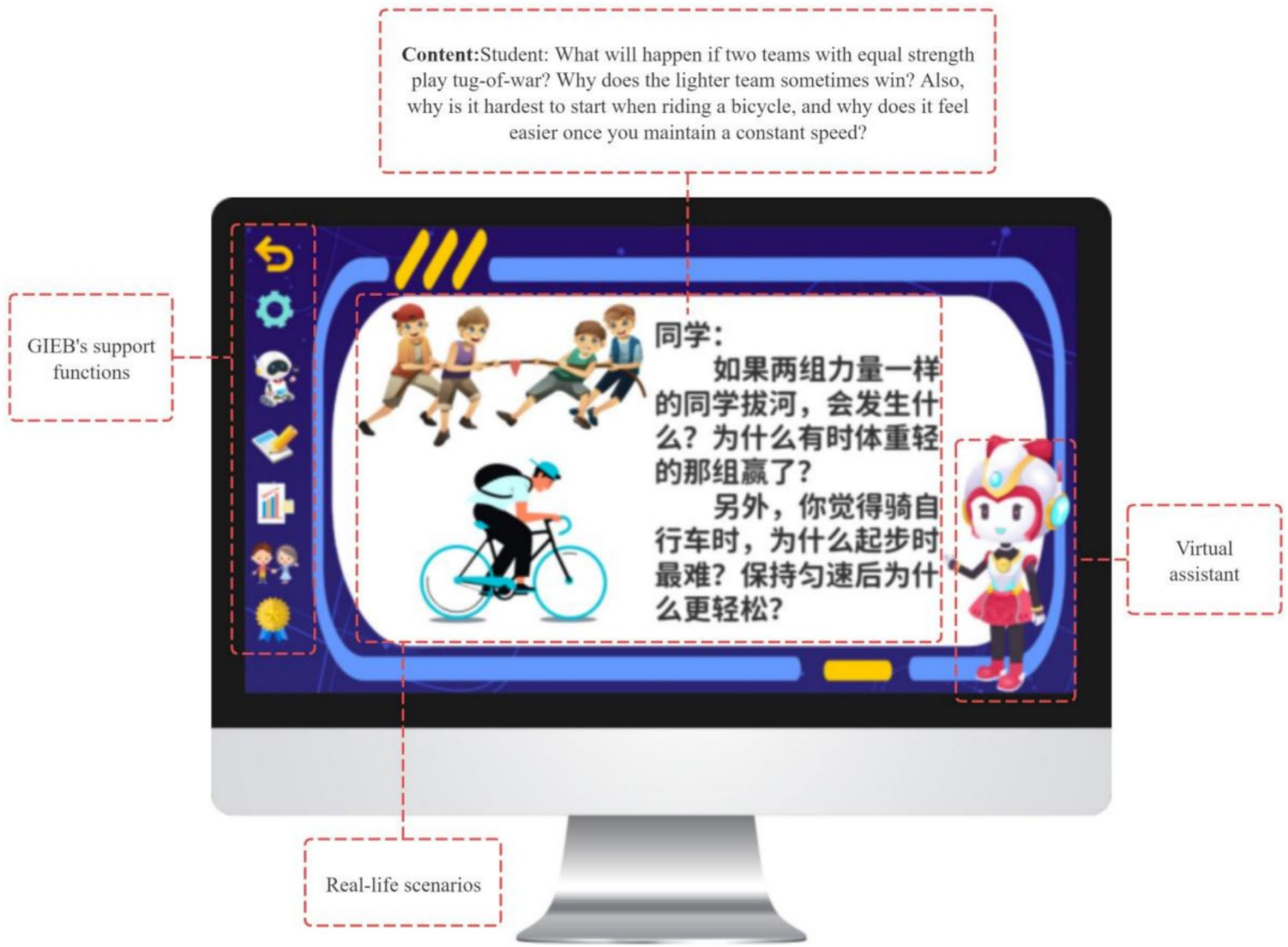
Everyday scenarios triggering prior conceptions and cognitive conflict.

The Gamified Interactive E-Book (GIEB) focuses on the “Force” unit of the Grade 8 physics curriculum in China, covering core topics such as the definition of force, its three elements, elastic force, and gravity. To initiate learning, the system employs everyday scenarios (e.g., “Why does cycling become harder the more you pedal?” or “Which team will win in a tug-of-war?”) to activate prior knowledge, elicit intuitive judgments, and introduce cognitive conflict, thereby preparing students for subsequent learning tasks (see Figure 5).

**Figure 6:**
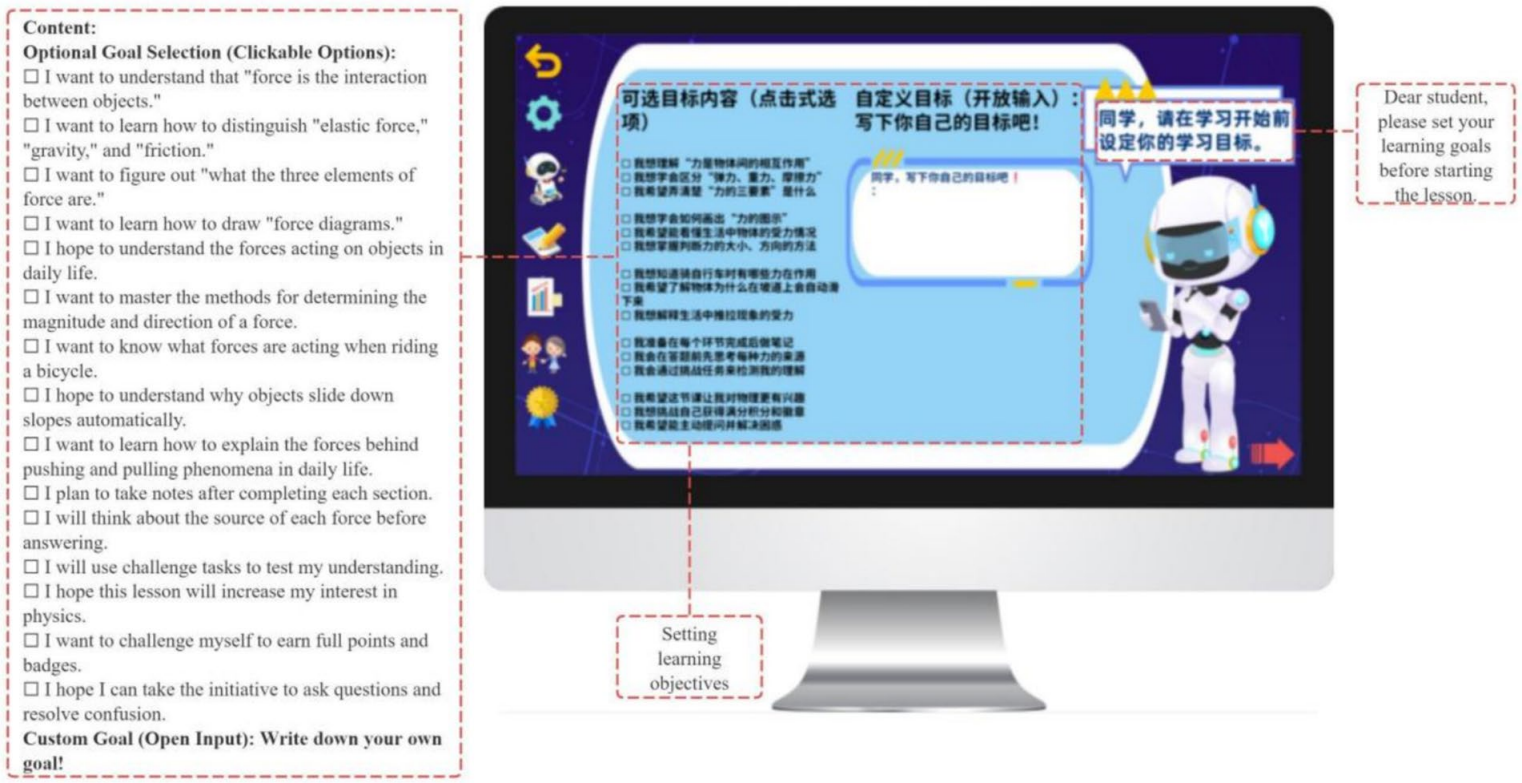
GIEB guides students to set their learning goals.

Building on this activation, the GIEB guides students to articulate explicit lesson objectives aligned with the Compulsory Education Physics Curriculum Standards (2022 edition), including understanding force as an interaction, mastering its elements and representations, identifying common forces, and applying concepts to real-world contexts (see Figure 6).

**Figure 7:**
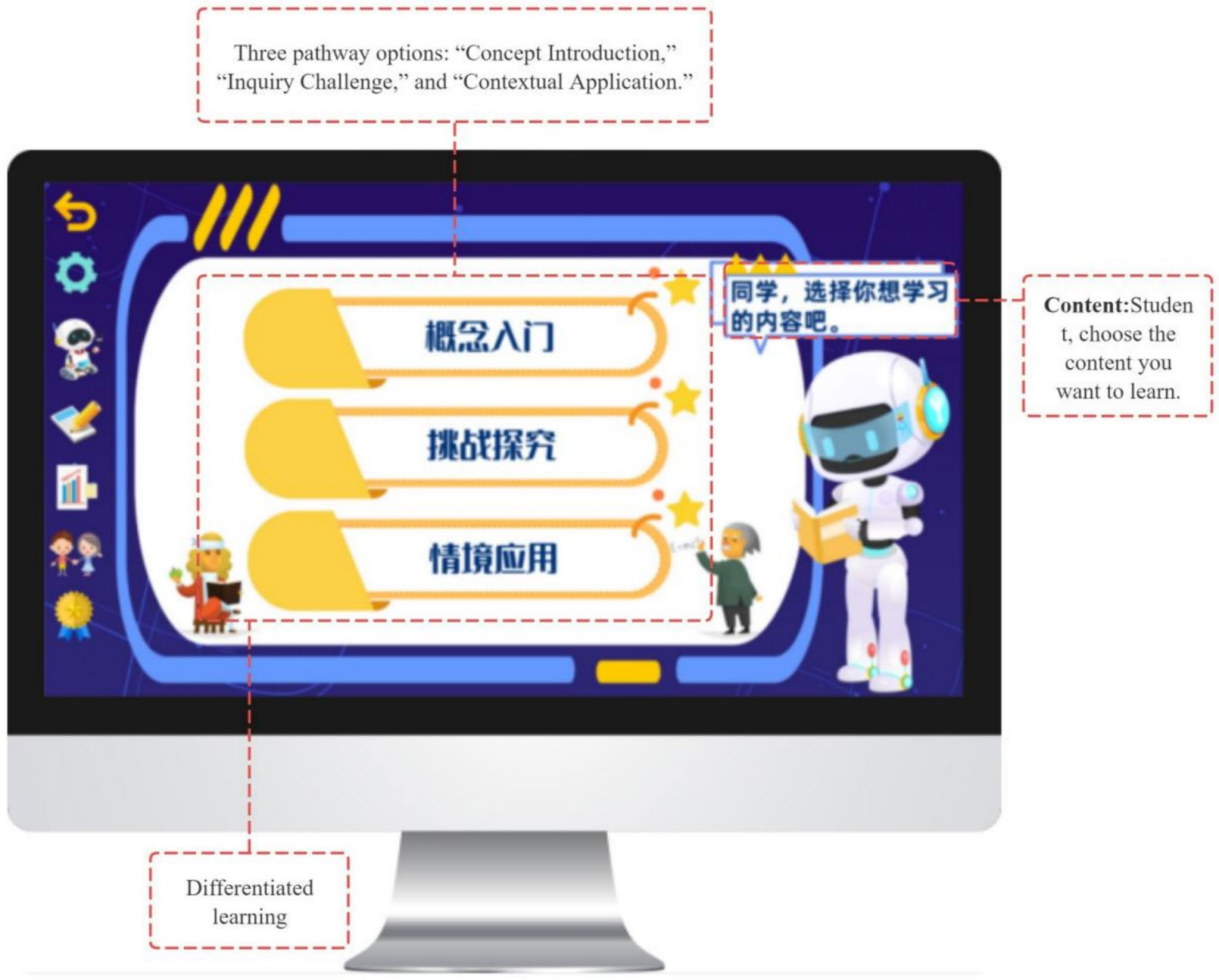
Three learning modes in GIEB: Concept introduction, challenge exploration, and contextual application.

**Figure 8:**
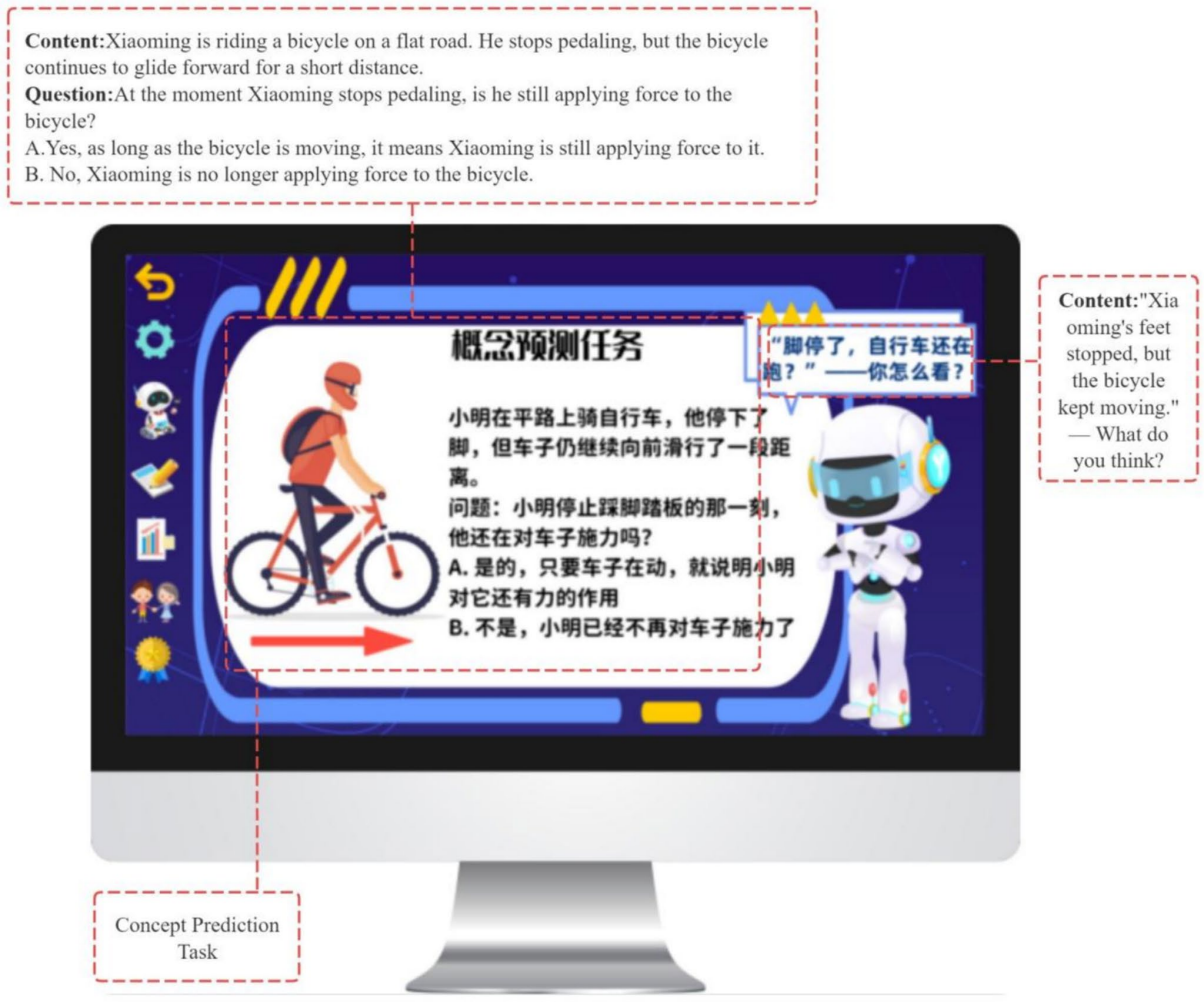
Concept prediction task.

**Figure 9:**
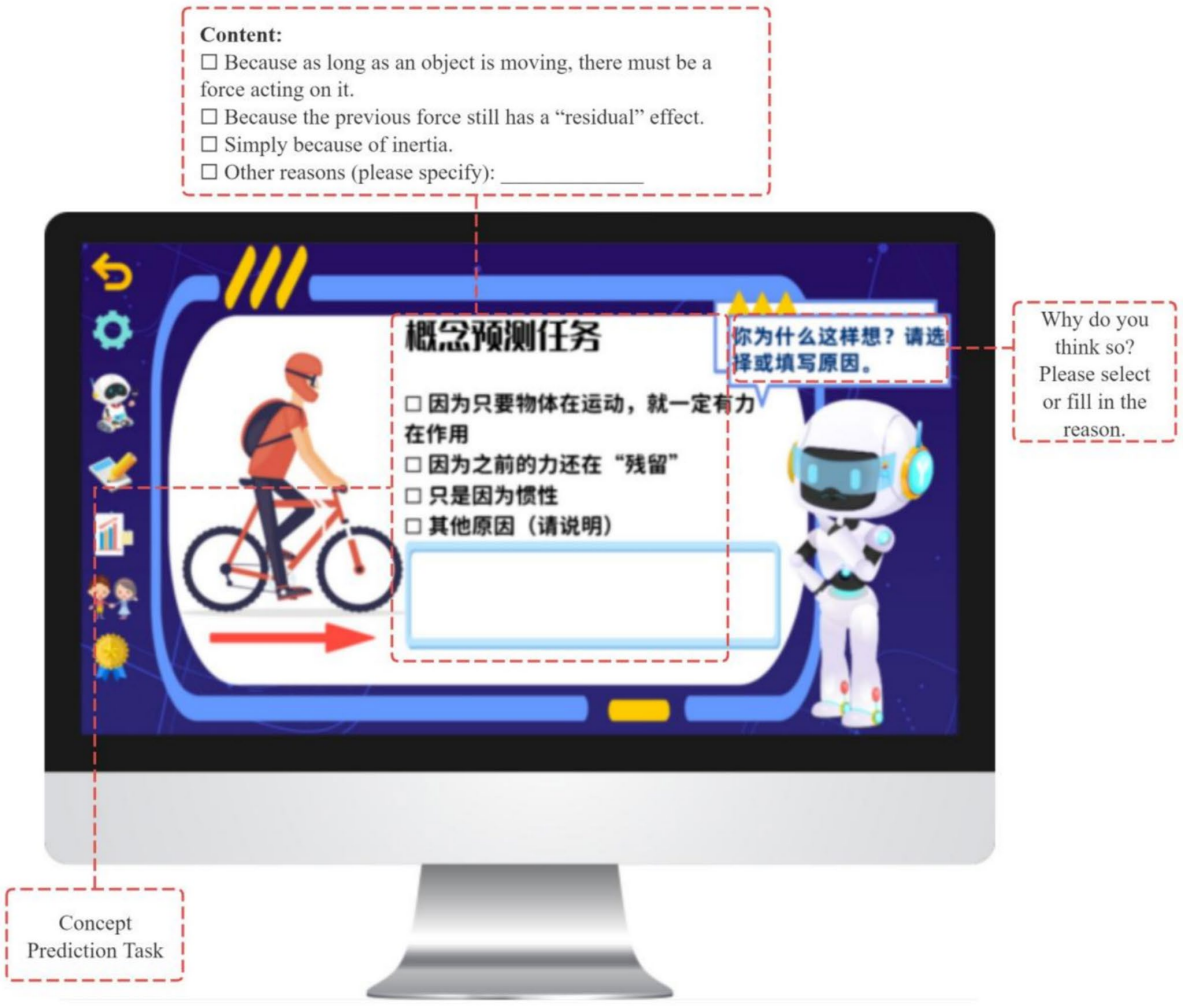
Concept prediction task.

To support goal-oriented planning, the system provides three engagement modes—Conceptual Introduction, Inquiry Challenge, and Contextual Application (see Figure 7)—each incorporating a shared concept prediction task adapted to the selected pathway (see Figures 8, 9). Students’ predictions are recorded and used as reference points for subsequent prediction–verification–reflection cycles, thereby operationalizing the forethought phase of Zimmerman’s SRL model.

**Figure 10:**
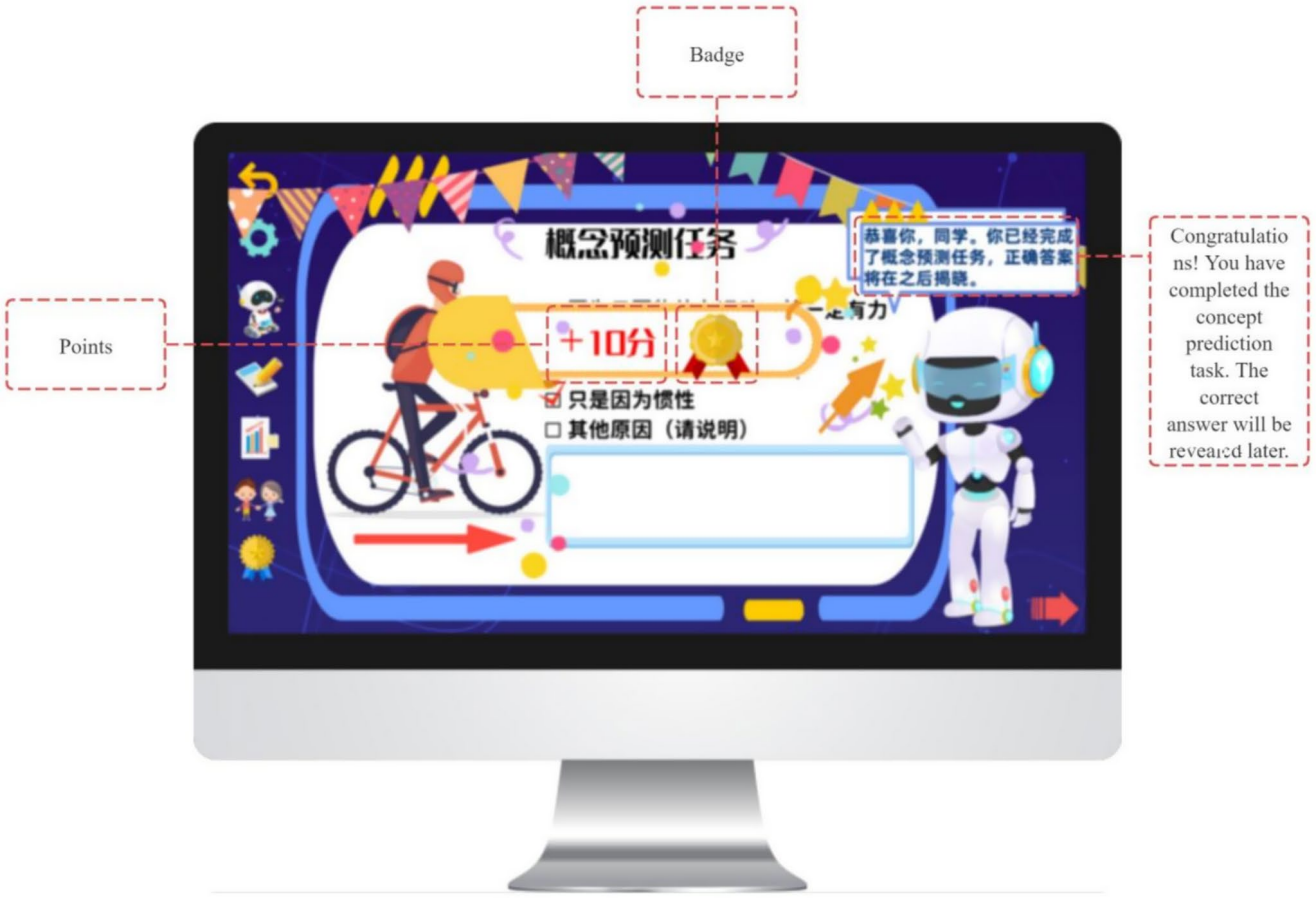
Instant motivational feedback provided by GIEB.

**Figure 11:**
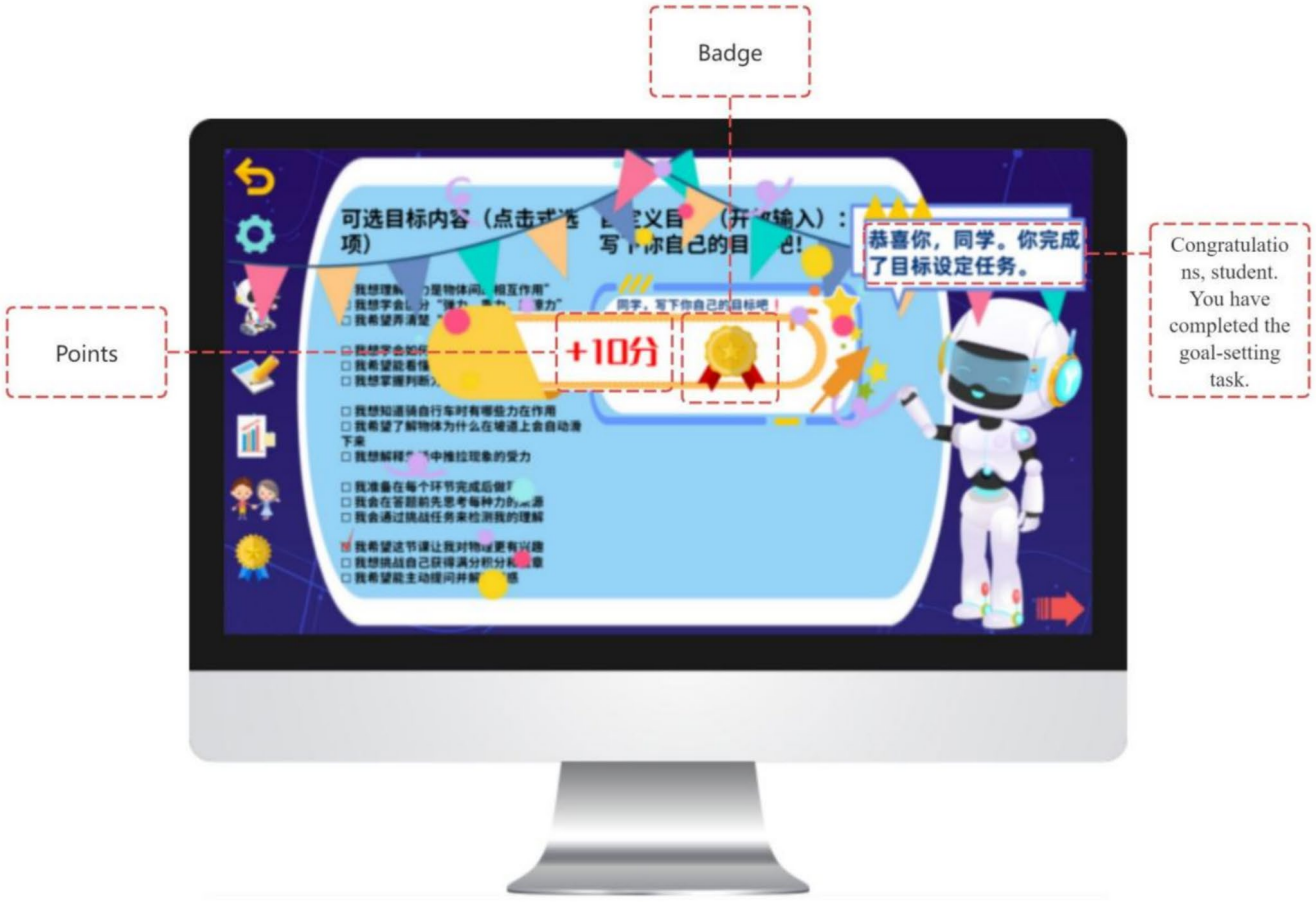
Instant motivational feedback provided by GIEB.

Gamification elements such as badges and points are provided following task completion as informational feedback, signaling progress and task engagement while preserving learner choice. In this way, the planning phase integrates metacognitive goal-setting with autonomy-supportive design principles derived from Self-Determination Theory (see Figures 10, 11).

#### 2.3.2 Monitoring phase: supporting strategy application and process regulation

During the monitoring phase, the GIEB supports students’ ongoing awareness of comprehension and strategy use, corresponding to the performance phase of Zimmerman’s SRL model. To this end, the system integrates three core scaffolding mechanisms: stepwise guided practice, error-specific prompts, and answer-tracking tools.

**Figure 12:**
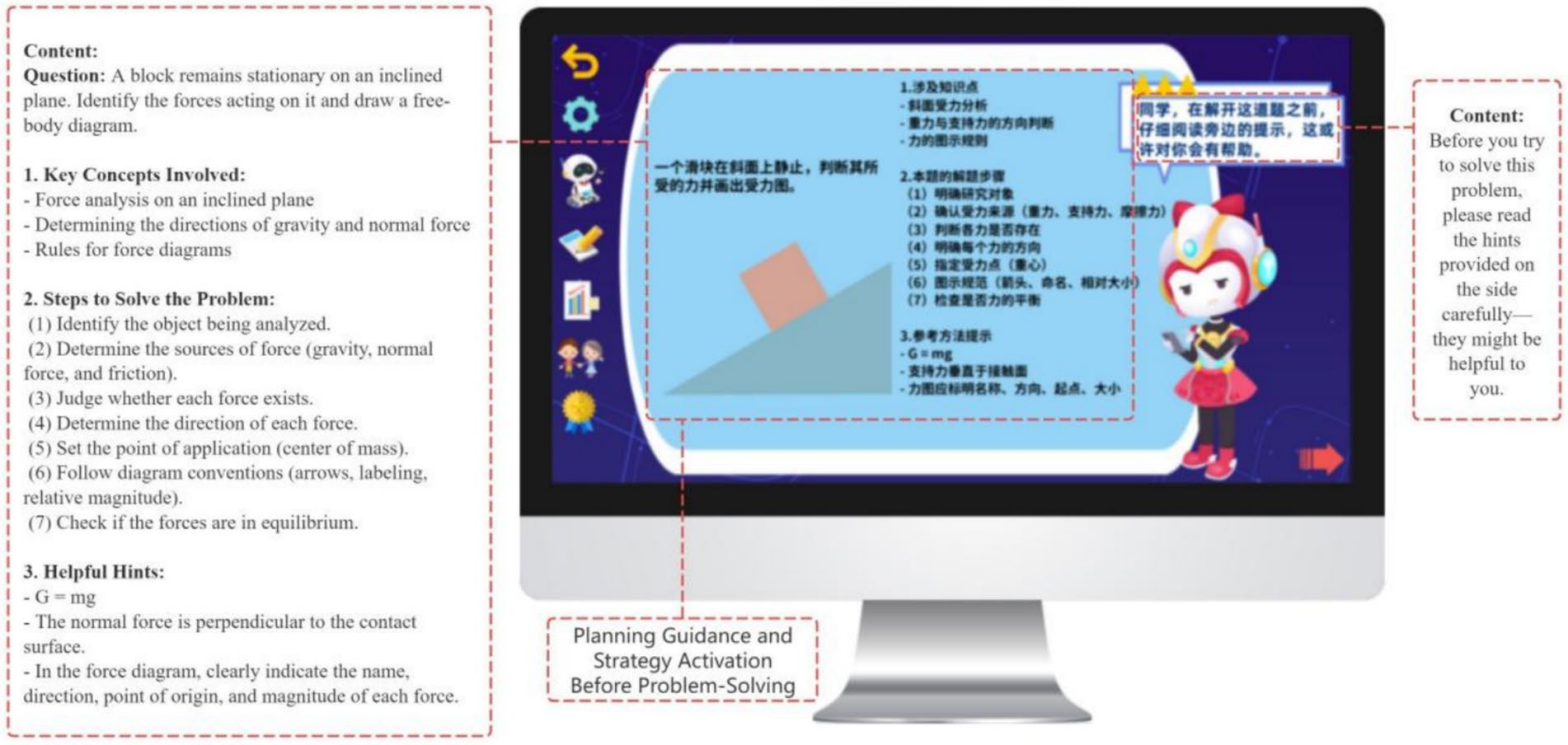
Guiding students to clarify problem-solving steps and key formulas.

**Figure 13:**
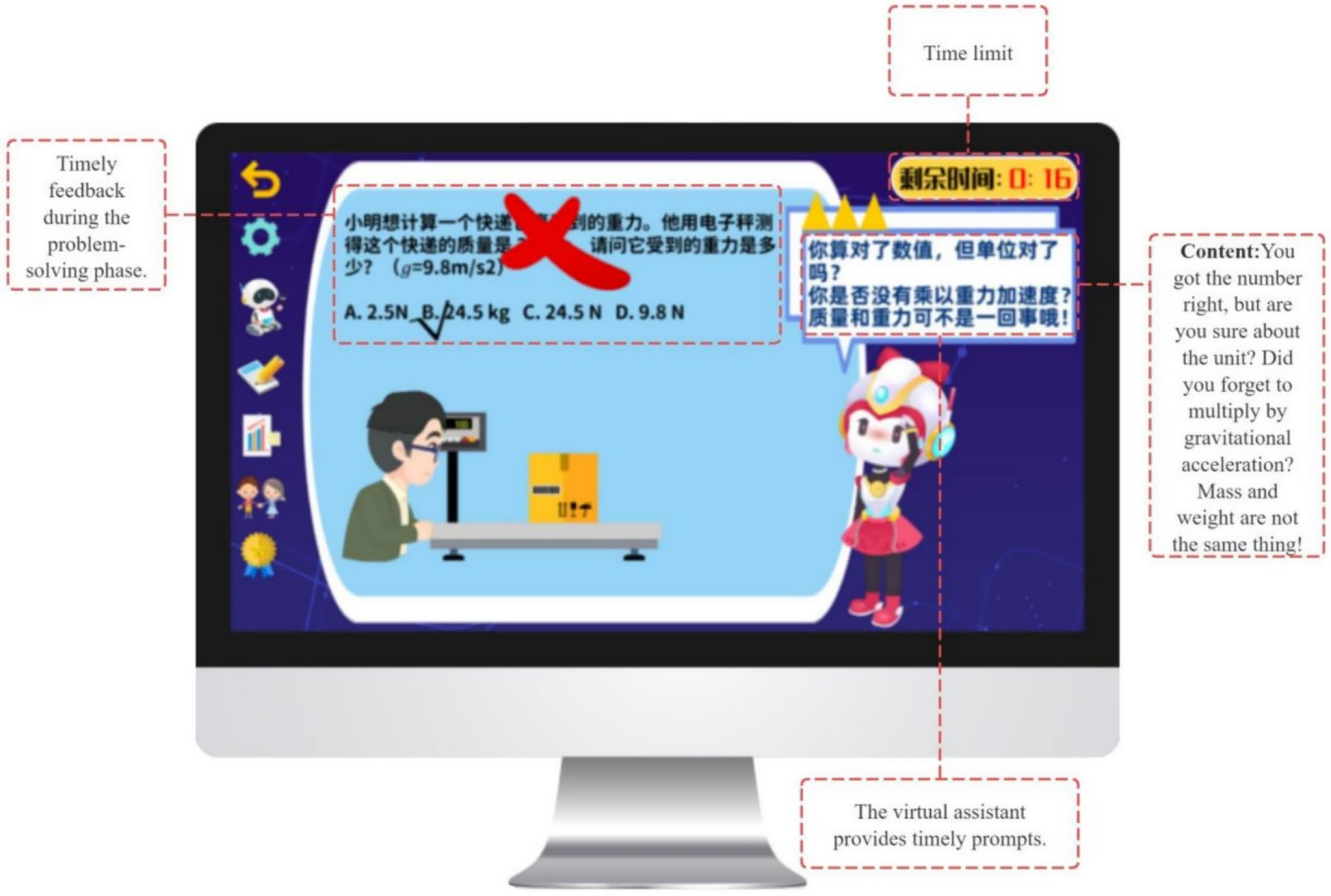
In-the-moment guidance during problem solving.

Each exercise set includes a “My Problem-Solving Approach” section that prompts students to externalize solution steps and key formulas prior to answering, thereby strengthening the linkage between planning and monitoring (Zimmerman, 2002) (see Figure 12). In addition, the system provides real-time feedback at critical problem-solving junctures. For example, prompts such as “Have you considered unit consistency?” guide learners to identify and correct cognitive deviations, forming a feedback-driven regulation mechanism (Butler and Winne, 1995) (see Figure 13).

The GIEB also generates visualized answer-tracking charts that display accuracy rates, error types, and learning trajectories across knowledge points. Functioning as a basic learning dashboard, this tool supports explicit monitoring and lays the foundation for subsequent reflection and strategy refinement.

Gamification mechanisms such as Focus Badges and Skill Points function as competence-related signals, highlighting effective engagement and strategy application without imposing controlling incentives, thereby supporting sustained task involvement.

#### 2.3.3 Evaluation and regulation phase: reflection, adjustment, and strategy enhancement

In the evaluation and regulation phase, the GIEB supports learners’ self-reflection and adaptive regulation, corresponding to the self-reflection phase of Zimmerman’s SRL model. This phase integrates a “prediction–verification–reflection” cycle (Tang and Kay, 2014) with data-driven regulatory support (Azevedo et al., 2010).

**Figure 14:**
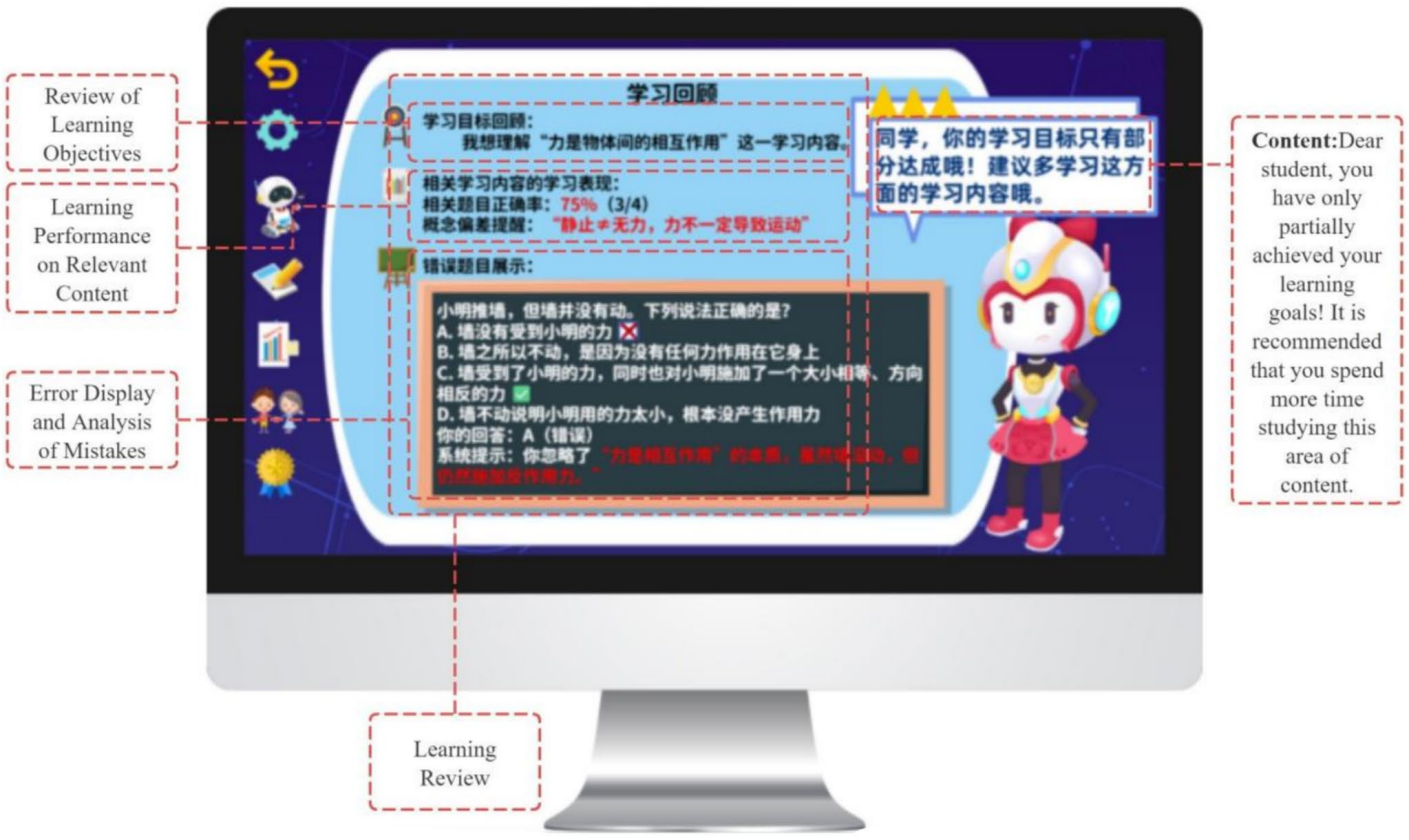
Learning Reflection Interface.

The system revisits initial learning goals and generates a “Goal Achievement Review” panel that juxtaposes students’ predictions with task performance, encouraging awareness of conceptual change and sources of error (see Figure 14). Reflection-oriented activities, such as Error Analysis and Reflection Cards, further scaffold students’ articulation of insights and translation of reflections into actionable strategy adjustments.

**Figure 15:**
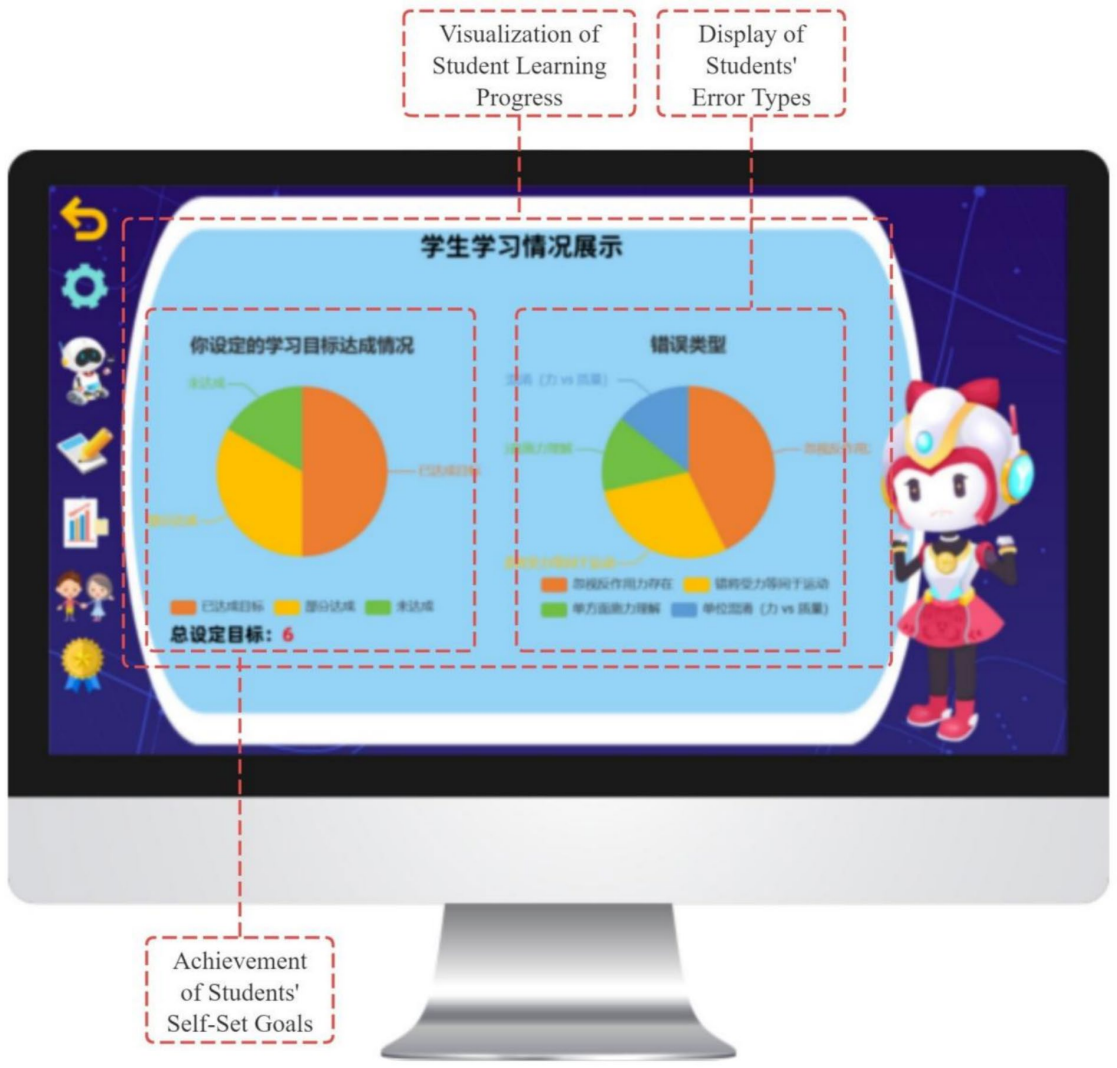
Dashboard for monitoring learning progress.

To support regulation, the Learning Dashboard visualizes behavioral trajectories, including accuracy patterns and error distributions, enhancing students’ awareness of learning processes and enabling strategy optimization (see Figure 15). Based on reflection outcomes, the system provides adaptive task recommendations (e.g., review or extended challenge pathways). Badges, points, and performance displays serve as informational feedback that highlights regulatory behaviors and learning progress, while leaderboard mechanisms support social visibility and a sense of relatedness without constraining learner autonomy.

Table 1 summarizes the key functions of the GIEB and how they were implemented using the Yoya platform:

**Table 1:** Key functions of the GIEB.

**Function**	**Description**	**Yoya’s role**
Self-directed learning and path selection	Providing three learning modes (Concept Introduction, Challenge Inquiry, and Contextual Application) along with prediction tasks to trigger cognitive conflict and support personalized path selection.	Managing differentiated learning paths and auto-generating prediction tasks to support content retrieval and student choice tracking.
Goal setting and strategy activation	Present learning objectives and guide students to complete “problem-solving plans” and reflection cards to strengthen planning-oriented thinking and strategy awareness.	Embed structured input prompts to record goals, problem-solving steps, and reflection content, supporting pre-, during-, and post-task tracking and comparison.
Process prompts and immediate feedback	The virtual assistant provides prompts at key steps, and the system automatically generates error-based feedback after each response to guide students in adjusting their strategies.	Integrating a rule engine and feedback templates enables contextualized prompts and performance-based personalized feedback delivery.
Data tracking and visualization	Real-time recording and visualization of task performance (e.g., accuracy rate, error types, task duration) are used to build a learning dashboard that supports student reflection and instructional intervention.	Implement behavior data collection, chart generation, and dashboard display to support data export and systematic analysis.
Intelligent recommendation and relearning support	Based on students’ learning performance, the system intelligently recommends “review and reinforcement” or “extended challenge” tasks to support personalized regulation pathways and strategy transfer.	By embedding recommendation algorithms and a content repository, the system enables dynamic re-learning task delivery and individualized intervention.
Incentive mechanisms and social connectedness	Enhance students’ sense of competence, autonomy, and social belonging through points, badges, leaderboards, and other mechanisms.	Configure diversified incentive mechanisms and leaderboard systems to support learning outcome display, virtual interaction, and social sharing.

### 2.4 Game elements in GIEB

The GIEB incorporates a systematic gamification design to support students’ engagement with metacognitive self-regulation strategies during physics learning. Prior research suggests that well-designed gamification elements, when embedded within strategy-based learning environments, can support learners’ engagement, strategy use, and academic performance (Chen C. et al., 2024; Ateş and Polat, 2025; Al-Hosni et al., 2024). In the present study, core game elements are integrated across the phases of metacognitive self-regulation—planning, monitoring, evaluation, and regulation—to provide structured support for cognitive regulation and strategic reflection.

The design of the GIEB follows the gamification framework proposed by Kam and Umar (2018), informed by Self-Determination Theory (SDT). Within this framework, gamification elements are not treated as direct drivers of intrinsic motivation but as design features intended to support autonomy, competence, and relatedness in an autonomy-supportive manner.

Autonomy support is realized by allowing students to control their learning pathways and pace. Learners may start, pause, repeat, or revisit content freely, and retry tasks without penalty. Based on learning feedback, students can choose between “review and consolidation” and “extended challenge” pathways. Visual dashboards further enable learners to monitor their progress and make informed decisions about subsequent learning activities (Chen C. et al., 2024).

Competence facilitation is supported through multi-level problem-solving tasks, structured prompts, and immediate explanatory feedback. Points and badges are provided as informational signals of task completion, progress, and effective strategy use, rather than as controlling rewards. Incorrect responses trigger diagnostic feedback to help learners identify and correct misconceptions, while a virtual assistant offers metacognitive prompts that reinforce strategy awareness. Time-limited challenges and adaptive task recommendations are used to align task difficulty with learners’ performance trajectories (Azevedo et al., 2010).

Relatedness development is addressed through social visibility and interaction mechanisms. The GIEB allows learners to display achievements, view leaderboards, and receive peer recognition through achievement walls. In addition, the virtual assistant provides contextualized encouragement during key learning moments, supporting a sense of social presence. Community features such as likes, comments, and peer congratulations further promote emotional support and strategy exchange among learners.

In summary, the GIEB integrates gamification elements with metacognitive self-regulation strategy training to construct a learning environment that emphasizes autonomy-supportive design, competence-related feedback, and social visibility. Rather than relying on reward-driven motivation, the gamification mechanisms function as scaffolds that support students’ regulation of learning and sustained engagement in junior high school physics.

Table 2 summarizes the gamification elements applied in the GIEB, based on autonomy, competence, and relatedness.

**Table 2:** Gamification elements applied in the GIEB.

**SDT psychological need**	**Gamification elements applied in the GIEB**	**Purpose and function**
Autonomy	- Freedom to control learning path and pace (start, pause, skip, repeat) - No-penalty mechanism for repeated task attempts - Autonomous selection between “Review & Reinforcement” or “Extended Challenge” paths - Visual learning dashboard for progress tracking	Supports learners in autonomously planning, executing, and regulating learning activities based on their cognitive states, thereby enhancing their sense of control and engagement in learning (Chen et al., 2024a; Buckley and Doyle, 2017).
Competence	- Tiered, strategic problem-solving tasks - Virtual assistant providing structured prompts and problem-solving strategy guidance - Points and badge system for correct answers - Explanatory feedback triggered by incorrect responses - Time limit mechanism simulating real-world problem-solving scenarios - Intelligent task recommendation system	Providing immediate feedback and challenge support within structured problem-solving and self-regulatory processes helps foster students’ perceived competence and sense of mastery (Deci and Ryan, 2000; Azevedo et al., 2010).
Relatedness	- Achievement sharing features (points and badges) - Leaderboards and achievement display wall - Contextualized encouragement and feedback from the virtual assistant - Community features (likes, comments, congratulations)	Foster emotional connections among learners, peers, the system, and learning tasks by embedding social interaction within learning and feedback activities, thereby supporting a sense of belonging (Deci and Ryan, 2000).

To further clarify how gamification elements in the GIEB are operationalized in relation to the core components of metacognitive self-regulation, Table **3** presents a detailed mapping across the three phases of self-regulated learning—planning, monitoring, and evaluation/regulation. Each phase is linked to specific gamification mechanisms and their corresponding operational implementations within the GIEB, illustrating how the system design translates theoretical constructs into concrete learning supports.

**Table 3:** Mapping of gamification elements to metacognitive self-regulation phases in the GIEB.

**SRL phase**	**Key self-regulation process**	**Gamification element**	**Operationalization in GIEB**
Planning (Forethought)	Goal setting and strategic planning	Everyday scenario activation; Learning path selection; Goal-setting interface; Concept prediction tasks; Badges and points as informational feedback	The system employs everyday scenarios (e.g., “Why does cycling become harder the more you pedal?”) to activate prior knowledge and introduce cognitive conflict. Students then articulate explicit lesson objectives aligned with curriculum standards and choose among three engagement modes (Conceptual Introduction, Inquiry Challenge, Contextual Application). Each pathway incorporates a concept prediction task; predictions are recorded as reference points for subsequent prediction–verification–reflection cycles, operationalizing the forethought phase. Badges and points are awarded upon task completion as informational feedback signaling progress while preserving learner choice.
Monitoring (Performance)	Self-observation, strategy application, and comprehension tracking	“My Problem-Solving Approach” prompts; Error-specific real-time feedback; Visualized answer-tracking charts; Focus Badges and Skill Points	Each exercise set includes a “My Problem-Solving Approach” section prompting students to externalize solution steps and key formulas prior to answering. The system provides real-time feedback at critical problem-solving junctures (e.g., “Have you considered unit consistency?”) to guide identification and correction of cognitive deviations. Visualized answer-tracking charts display accuracy rates, error types, and learning trajectories across knowledge points, functioning as a basic learning dashboard. Focus Badges and Skill Points serve as competence-related signals highlighting effective engagement and strategy application.
Evaluation/Regulation (Self-Reflection)	Reflective evaluation, causal attribution, and strategy adjustment	Goal Achievement Review panel; Error Analysis and Reflection Cards; Learning Dashboard; Adaptive task recommendations; Badges, points, and performance displays; Leaderboard and achievement sharing; Virtual assistant encouragement	The system revisits initial learning goals and generates a Goal Achievement Review panel juxtaposing students’ predictions with task performance, encouraging awareness of conceptual change and sources of error. Error Analysis and Reflection Cards scaffold articulation of insights and translation of reflections into actionable strategy adjustments. The Learning Dashboard visualizes behavioral trajectories including accuracy patterns and error distributions, supporting strategy optimization. Based on reflection outcomes, the system provides adaptive task recommendations (review or extended challenge pathways). Badges, points, and performance displays serve as informational feedback highlighting regulatory behaviors. Leaderboard mechanisms and the virtual assistant’s contextualized encouragement support social visibility and relatedness without constraining autonomy.

## 3 Methods

### 3.1 Research design

This study employed a quasi-experimental pretest–posttest design to examine the effects of a Gamified Interactive E-Book (GIEB), which integrates metacognitive self-regulation strategies, on Chinese junior high school students’ learning motivation, metacognitive self-regulation ability, and physics achievement. Two intact classes were randomly designated as the experimental group and the control group respectively—a class-level assignment necessitated by the practical constraints of school-based research, where individual-level randomization would disrupt established classroom structures and risk treatment contamination across conditions (Campbell and Stanley, 1963). Students were not informed of their group allocation in order to minimize potential Hawthorne effects. This class-level assignment ensured that all students within each class followed the same instructional approach—the GIEB group engaged exclusively with the gamified interactive e-book, while the TL group received conventional lecture-based instruction throughout—thereby preventing any sense of differential treatment or discrimination among students within the same classroom. It should be noted that, as a quasi-experimental design using intact classes, this study cannot fully rule out potential confounds associated with pre-existing between-class differences, such as classroom culture, peer dynamics, or prior teacher–student interaction patterns. The experimental group received instruction through the GIEB intervention, while the control group received traditional lecture-based instruction in which the same teacher orally introduced metacognitive self-regulation strategies (i.e., planning, monitoring, and evaluation) as part of regular classroom teaching, without the support of digital tools, structured scaffolding, immediate feedback systems, or gamification elements.

Both groups completed pretests and posttests to measure the target variables. In the data analysis phase, pretest scores were included as covariates to control for baseline differences and to enhance the interpretive validity of the findings.

### 3.2 Participants

The sample for this study was drawn from two Grade 8 classes at a public junior high school in Hefei, Anhui Province, China, comprising a total of 60 students aged 13–14 years. The school was selected through convenience sampling. Prior to the commencement of the study, informed consent forms were obtained from the parents or legal guardians of all participants. Students were also explicitly informed of their right to withdraw from the study at any stage without penalty. The two classes were then randomly designated as the experimental group (n = 30) and the control group (n = 30).

To ensure sufficient statistical power, *a priori* power analysis was conducted during the design phase using G*Power software. The analysis was based on an ANCOVA model with a statistical power of 0.80, a significance level of *α* = 0.05, and an effect size of *f* = 0.4. According to Cohen’s (1988) guidelines, *f* = 0.4 represents a large effect size, which is appropriate in contexts where interventions are expected to yield substantial impacts on learning outcomes.

The choice of *f* = 0.4 was justified on two grounds. First, Cohen’s (1988) effect size conventions are widely applied in educational intervention research, particularly in scenarios where interventions are hypothesized to strongly influence learning motivation and academic achievement. Second, prior empirical studies and meta-analyses have indicated robust effects of gamified learning and virtual laboratory interventions on student performance. For example, Subhash and Cudney’s (2018) meta-analysis reported that gamification significantly improves students’ motivation and achievement, with effect sizes ranging from *f* = 0.25 to *f* = 0.4.

Additional studies have also confirmed the positive effects of gamification strategies on learning motivation, metacognitive self-regulation, and academic performance (Chen C. et al., 2024; Ateş and Polat, 2025; Al-Hosni et al., 2024). Meta-analyses by Li et al. (2023) and Zeng and Sun (2023) further reported overall effect sizes of *g* = 0.822 and *g* = 0.782, respectively, both of which fall within the large-effect range.

Based on these parameters and supporting evidence, the power analysis indicated that a minimum of 52 students (26 per group) would be required to achieve adequate statistical power. The final sample of 60 students exceeded this minimum threshold. It should be acknowledged that the assumed effect size of f = 0.4 represents an optimistic estimate; under a more conservative assumption of *f* = 0.25 (medium effect), the required sample size would increase to approximately 128 participants. The present sample size of 60 therefore constitutes a limitation in detecting smaller effects. Nevertheless, the sample size is comparable to those reported in similar classroom-based quasi-experimental studies in educational technology research (e.g., Chen C. et al., 2024, *n* = 69; Al-Hosni et al., 2024, *n* = 60), where practical constraints of school-based interventions typically limit participant availability.

### 3.3 Research instruments

This study employed three primary instruments: a Junior High School Physics Learning Motivation Scale, a Metacognitive Self-Regulation Ability Scale, and a Physics Achievement Test.

#### 3.3.1 Junior high school physics learning motivation scale

The Junior High School Physics Learning Motivation Scale used in this study was adapted from the Science Motivation Questionnaire II (SMQ-II) developed by Glynn et al. (2011). The scale was revised to better align with the physics domain and the cognitive and developmental characteristics of junior high school students, with simplified wording to enhance comprehension.

The original SMQ-II consists of 25 items across five dimensions: intrinsic motivation, self-efficacy, self-determination, grade motivation, and career motivation, rated on a five-point Likert scale (1 = strongly disagree to 5 = strongly agree). In the original validation study, Glynn et al. reported excellent internal consistency (overall Cronbach’s *α* > 0.90; subscale α ranging from 0.82 to 0.91), and strong construct validity based on prior psychometric evaluations.

For the present study, one item (“I will use physics problem-solving skills in my career”) was removed to account for the developmental stage of junior high school students, resulting in a 24-item scale. The revised scale retained the original five-dimensional structure and scoring method, while language simplification improved age appropriateness. Example items include:

- Item 1: “The physics I learn is relevant to my life” (intrinsic motivation);
- Item 6: “I like to do better than other students on physics tests” (grade motivation);
- Item 11: “I am confident I will do well on physics tests” (self-efficacy);
- Item 16: “I am willing to put in the effort to learn physics” (self-determination);
- Item 21: “Learning physics will help me get a good job in the future” (career motivation).

Given the relatively small sample size in the present study, confirmatory factor analysis was not conducted. Instead, internal consistency reliability was assessed. The adapted scale demonstrated acceptable reliability, with an overall Cronbach’s α coefficient of 0.801, supporting its use with junior high school students.

#### 3.3.2 Metacognitive self-regulation scale

Metacognitive self-regulation ability was measured using the Metacognitive Self-Regulation subscale of the Motivated Strategies for Learning Questionnaire (MSLQ) developed by Pintrich et al. (1991). The subscale consists of 12 items assessing students’ use of planning, monitoring, and regulation strategies, rated on a five-point Likert scale (1 = strongly disagree to 5 = strongly agree).

The original MSLQ validation study reported satisfactory internal consistency for the metacognitive self-regulation subscale (Cronbach’s α = 0.79). Subsequent large-scale empirical studies and meta-analyses (e.g., Credé and Phillips, 2011; Tock and Moxley, 2017) have further demonstrated the scale’s construct validity and its consistent associations with academic achievement, learning engagement, and self-regulated learning behaviors across educational contexts.

The adapted version used in the present study retained all 12 items. Example items include:

- Item 1: “Before I begin studying, I think about the tasks I will need to complete and the goals I want to reach” (planning);
- Item 6: “When studying physics, I often check my level of understanding” (monitoring);
- Item 11: “When I feel my learning efficiency is low, I will adopt a new way of learning” (regulation).

In the present sample of junior high school students, the scale demonstrated strong internal consistency, with a Cronbach’s *α* coefficient of 0.832, indicating satisfactory reliability for this educational stage.

#### 3.3.3 Physics achievement test

The Physics Achievement Tests employed in this study were developed based on the “Force” unit from the Grade 8 physics textbook (People’s Education Press edition), which is widely used in China’s compulsory education system. This unit holds a central position within the junior high school physics curriculum, serving as a foundation for understanding mechanics. It covers key knowledge points such as the definition of force, the three elements of force, force diagrams, elastic force, and gravity, and provides the cognitive basis for subsequent topics including friction and Newton’s First Law.

To ensure alignment between the tests and the instructional content as well as to establish structural validity, the items were designed with reference to the textbook, supplementary teaching materials, and the official curriculum standards. The tests focused on the following core areas:

1. Definition and essence of force (i.e., force as an interaction between objects).
2. The three elements of force and their graphical representations (including acting and receiving objects, magnitude, direction, and point of application).
3. Common types of forces, their conditions, and characteristics, with particular emphasis on the identification and calculation of gravity and elastic force.
4. Problem analysis and force diagram construction based on real-life scenarios.

The draft tests were reviewed by three experienced junior high school physics teachers, each holding senior professional titles and over 10 years of teaching experience. The experts evaluated the scientific accuracy, clarity of wording, appropriateness of difficulty, and alignment with instructional objectives, and provided feedback for revision. The tests were subsequently refined to improve their content validity.

Each final version comprised four multiple-choice items and four structured short-answer items (including fill-in-the-blank and diagram-drawing tasks), with a total score of 80 points. The tests were designed to comprehensively assess students’ understanding of the “Force” unit, their ability to apply concepts, and their competence in analyzing real-world problems.

To further verify content validity, three senior teachers familiar with the Grade 8 physics syllabus independently evaluated the alignment between test items and learning objectives using a four-point Likert scale (1 = not relevant to 4 = highly relevant). The calculated average scale-level content validity index (S-CVI/Ave) for both tests was 1.0 (> 0.9), indicating excellent agreement and strong content validity (Polit and Beck, 2006).

Regarding reliability, it should be noted that internal consistency coefficients such as Cronbach’s α are designed for homogeneous scales measuring a single latent construct and are not appropriate for criterion-referenced achievement tests that deliberately sample across heterogeneous content domains (Tavakol and Dennick, 2011). The Physics Achievement Test in this study was designed to assess understanding across multiple distinct knowledge areas (e.g., definition of force, force diagrams, elastic force, gravity), and therefore content validity—rather than internal consistency—was prioritized as the primary indicator of measurement quality. The S-CVI/Ave of 1.0 reported above confirms strong content alignment with instructional objectives. Additionally, the use of parallel test forms for pretest and posttest, with equivalent content coverage, structure, and difficulty level as verified by expert review, provides further evidence of measurement consistency. Nevertheless, the limited number of items (eight per test) represents a constraint on measurement precision, and future studies should consider employing longer instruments or adopting item response theory approaches to improve the reliability and diagnostic sensitivity of achievement assessments.

### 3.4 Experimental procedure

**Figure 16:**
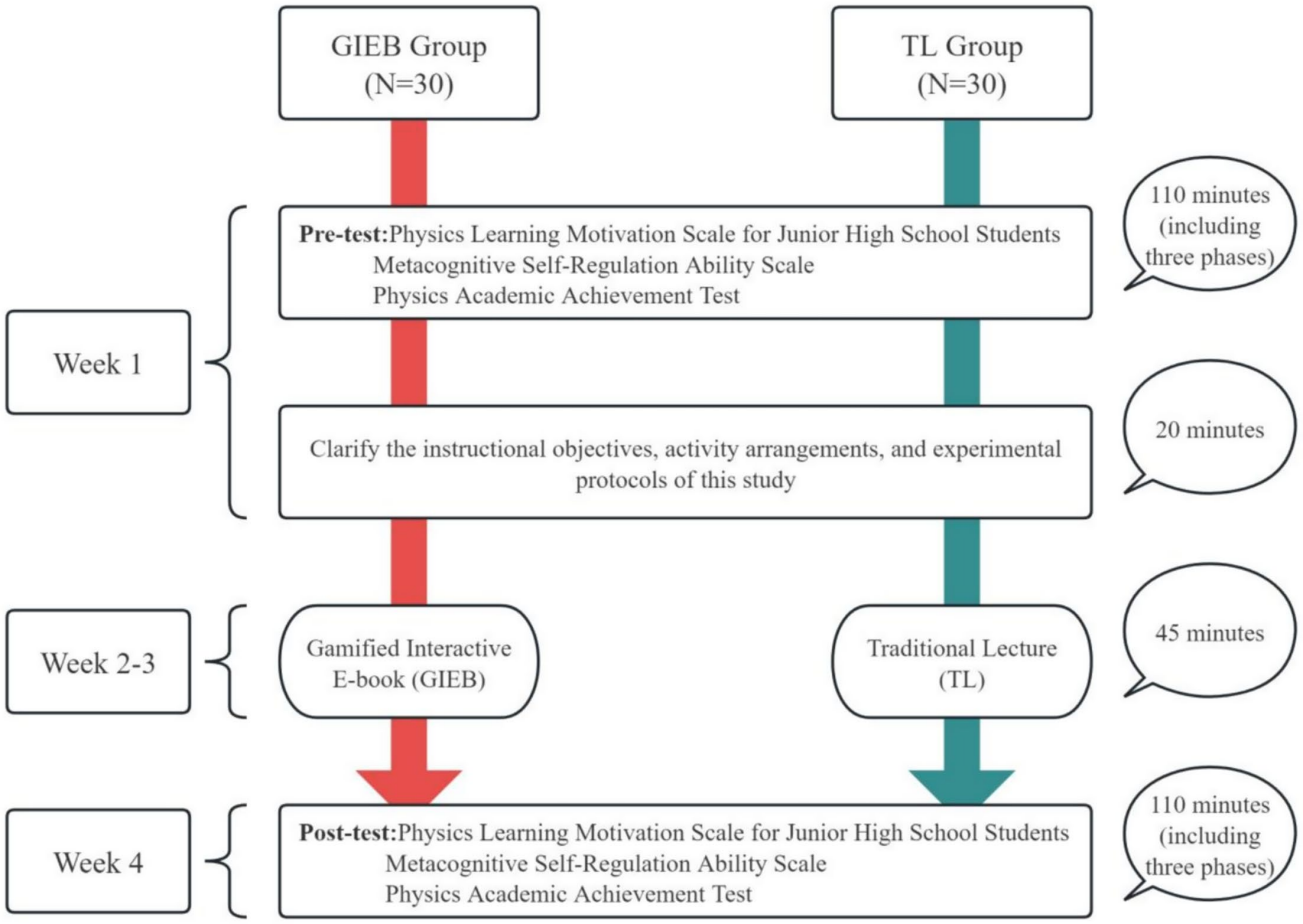
Experimental procedure.

The experiment lasted for 4 weeks, with instructional arrangements for the GIEB group (Gamified Interactive E-Book) and the TL group (Traditional Lecture) presented in Figure 16.

Prior to the intervention, all students completed a pretest to assess their baseline levels of physics learning motivation, metacognitive self-regulation ability, and physics achievement. The pretest lasted 110 min and was administered in three stages, using the following instruments: the Junior High School Physics Learning Motivation Scale, the Metacognitive Self-Regulation Scale, and the Physics Achievement Test.

Following the pretest, the teacher conducted a 20-min orientation session to explain the objectives, activity arrangements, and experimental protocols of the study. This ensured that participants had a clear understanding of the learning tasks and procedural requirements.

The intervention phase took place during Weeks 2 and 3. Instruction for both groups was delivered by the same teacher to ensure consistency. The GIEB group engaged with the interactive e-book that integrated metacognitive strategy prompts and gamification mechanisms, with each lesson lasting 45 min. The TL group, in contrast, received traditional lecture-based instruction covering the same physics content, with the teacher orally introducing metacognitive self-regulation strategies at relevant points during the lesson. Specifically, the teacher verbally reminded students to set learning goals before each topic (planning), to check their understanding during problem-solving exercises (monitoring), and to review their performance after completing tasks (evaluating and regulating). These oral reminders followed the same planning–monitoring–evaluation sequence embedded in the GIEB but were delivered without structured scaffolding, digital visualization, immediate automated feedback, or gamification elements. Both groups received identical physics content, instructional duration, and learning materials, with the systematic difference being the mode of metacognitive strategy delivery and the presence of gamified, technology-enhanced scaffolding.

In Week 4, a posttest was administered, lasting the same duration as the pretest, to evaluate changes in students’ learning motivation, metacognitive self-regulation ability, and physics achievement. To assess the psychological constructs, the identical Learning Motivation and Metacognitive Self-Regulation questionnaires were administered. However, for academic achievement, a parallel version of the Physics Achievement Test—equivalent in content, structure, and difficulty to the pretest—was employed. This design ensured the comparability of data while mitigating potential memory effects, thereby strengthening the reliability and validity of the research findings.

## 4 Data analysis and results

To compare the effectiveness of the Gamified Interactive E-Book (GIEB) intervention with the traditional lecture method, this study employed analysis of covariance (ANCOVA). ANCOVA was selected because it allows the inclusion of covariates to reduce error variance and enhance the accuracy of group comparisons. By statistically adjusting for initial differences between groups, ANCOVA also increases the reliability of the results. In this study, ANCOVA was applied to examine the differences between the experimental and control groups on posttest scores, with pretest scores included as covariates to control for pre-existing differences. This approach improved the precision and sensitivity of the analysis, ensuring a more accurate evaluation of the intervention effects.

To assess the impact of the GIEB compared with the traditional lecture method on students’ learning motivation, metacognitive self-regulation ability, and physics achievement, Eta squared (η^2^) was calculated as a measure of effect size. The use of effect size reduces the influence of sample size on outcomes and provides a more meaningful interpretation of the practical significance of results beyond mere statistical significance.

Prior to conducting ANCOVA, several assumptions were tested to ensure the validity of the analysis, including: (1) normality of data distribution, (2) homogeneity of variance, and (3) homogeneity of regression slopes. All statistical analyses were conducted using SPSS version 26, with the level of significance set at *p* < 0.05. To further verify the robustness of the results, independent-samples *t*-tests were conducted to compare the pretest scores of the two groups, confirming that no significant differences existed between groups prior to the intervention.

### 4.1 The impact of GIEB on students’ learning motivation

#### 4.1.1 Data analysis for learning motivation

To examine the impact of the Gamified Interactive E-Book (GIEB) on students’ learning motivation, a series of statistical analyses was conducted.

First, the Shapiro–Wilk test was performed to assess the normality of score distributions for both the experimental and control groups in the pretest and posttest of learning motivation. The results (*p* = 0.682, *p* = 0.909, *p* = 0.613, *p* = 0.902) indicated that all *p*-values were greater than 0.05, suggesting that the data met the assumption of normal distribution.

Second, Levene’s test of homogeneity of variances was applied to examine variance equality across the groups. The result (*p* = 0.773 > 0.05) confirmed that the homogeneity of variance assumption was satisfied. In addition, the test of homogeneity of regression slopes yielded *F* = 0.034, *p* = 0.855 > 0.05, further supporting that the assumptions required for ANCOVA were met.

Third, an independent-samples *t*-test was conducted to compare baseline differences between the two groups on pretest motivation scores. The result (*p* = 0.568 > 0.05) revealed no significant difference between the experimental and control groups at the pretest stage, confirming group comparability prior to the intervention (see Table 4).

**Table 4:** Independent samples *t*-test results for pretest scores between experimental and control groups.

Variable	Group	*N*	Mean	S.D	*t*	df	*p*
Pre-test	GIEB	30	72.433	14.374	−0.574	58	0.568
	TL	30	74.567	14.429			

#### 4.1.2 Findings on learning motivation

Finally, analysis of covariance (ANCOVA) was conducted to control for the influence of pretest scores and to examine the effect of the Gamified Interactive E-Book (GIEB) intervention on students’ posttest learning motivation. As shown in Table 5, the ANCOVA results revealed a significant difference between the two groups on adjusted posttest scores. The experimental group (GIEB) achieved a mean score of 89.446, which was significantly higher than that of the traditional lecture (TL) group (M = 75.820), *F*(1, 57) = 22.832, *p* < 0.001, η^2^ = 0.286.

**Table 5:** Descriptive data and ANCOVA results for the learning motivation posttest.

Variable	Group	*N*	Mean	S.D	adjusted Mean	*F*	*P*	η^2^
Post-test	GIEB	30	88.533	16.226	89.446	22.832	<0.001	0.286
	TL	30	76.733	16.706	75.820			

The effect size (η^2^ = 0.286) indicates a moderate-to-large magnitude of impact, suggesting that the GIEB intervention was considerably more effective in enhancing students’ learning motivation compared to the traditional lecture method.

### 4.2 The impact of GIEB on students’ metacognitive self-regulation ability

#### 4.2.1 Data analysis for metacognitive self-regulation ability

To examine the impact of the Gamified Interactive E-Book (GIEB) on students’ metacognitive self-regulation ability, a series of statistical analyses was conducted.

First, the Shapiro–Wilk test was performed to assess the normality of score distributions for both the experimental and control groups in the pretest and posttest. The results (*p* = 0.181, *p* = 0.384, *p* = 0.159, *p* = 0.130) indicated that all *p*-values were greater than 0.05, confirming that the data satisfied the assumption of normal distribution.

Second, Levene’s test of homogeneity of variances was conducted to examine variance equality across the groups. The result (*p* = 0.770 > 0.05) confirmed that the assumption of homogeneity of variances was met. Furthermore, the test of homogeneity of regression slopes yielded *F* = 1.489, *p* = 0.228 > 0.05, providing additional support that the assumptions required for ANCOVA were satisfied.

Finally, an independent-samples *t*-test was carried out to compare baseline differences between the two groups on pretest scores of metacognitive self-regulation ability. The result (*p* = 0.615 > 0.05) revealed no significant difference between the experimental and control groups, confirming that the two groups were comparable prior to the intervention (see Table 6).

**Table 6:** Independent samples t-test results for pretest scores between experimental and control groups.

Variable	Group	*N*	Mean	S.D	*t*	df	*p*
Pre-test	GIEB	30	36.333	10.094	−0.506	58	0.615
	TL	30	37.700	10.809			

#### 4.2.2 Findings on metacognitive self-regulation ability

Finally, analysis of covariance (ANCOVA) was performed to control for the influence of pretest scores and to examine the effect of the Gamified Interactive E-Book (GIEB) intervention on students’ posttest scores of metacognitive self-regulation ability. As presented in Table 7, the ANCOVA results revealed a significant difference between the two groups on adjusted posttest scores. The experimental group (GIEB) achieved a mean score of 47.973, which was significantly higher than that of the traditional lecture (TL) group (M = 40.361), *F*(1, 57) = 19.956, *p* < 0.001, η^2^ = 0.259.

**Table 7:** Descriptive data and ANCOVA results for the metacognitive self-regulation posttest.

Variable	Group	*N*	Mean	S.D	adjusted Mean	*F*	*P*	η^2^
Post-test	GIEB	30	47.700	7.003	47.973	19.956	<0.001	0.259
	TL	30	40.633	8.426	40.361			

The effect size (η^2^ = 0.259) indicates a moderate-to-large effect, suggesting that the GIEB intervention was substantially more effective than the traditional lecture method in enhancing students’ metacognitive self-regulation ability.

### 4.3 The impact of GIEB on students’ physics achievement

#### 4.3.1 Data analysis for physics achievement

To evaluate the impact of the Gamified Interactive E-Book (GIEB) on students’ physics achievement, a series of statistical analyses was conducted.

First, the Shapiro–Wilk test was used to assess the normality of score distributions for both the experimental and control groups in the pretest and posttest. The results (*p* = 0.656, *p* = 0.209, *p* = 0.083, *p* = 0.698) indicated that all *p*-values were greater than 0.05, confirming that the data satisfied the assumption of normal distribution.

Second, Levene’s test of homogeneity of variances was applied to evaluate equality of variance between the two groups. The result (*p* = 0.750 > 0.05) supported the assumption of homogeneity of variances. In addition, the test of homogeneity of regression slopes yielded *F*(1, 73) = 0.003, *p* = 0.956 > 0.05, further confirming that the assumption of homogeneity was met.

Finally, to verify baseline comparability prior to the intervention, an independent-samples *t*-test was conducted. The result (*p* = 0.863 > 0.05) indicated no significant difference between the two groups on pretest scores, demonstrating that the groups were comparable before the intervention (see Table 8).

**Table 8:** Independent samples t-test results for pretest scores between experimental and control groups.

Variable	Group	*N*	Mean	S.D	*t*	df	*p*
Pre-test	GIEB	30	39.000	7.129	−0.173	58	0.863
	TL	30	39.333	7.774			

Taken together, these results confirmed that all assumptions required for conducting analysis of covariance (ANCOVA) were satisfied.

#### 4.3.2 Findings on physics achievement

Finally, analysis of covariance (ANCOVA) was conducted to examine the effect of the Gamified Interactive E-Book (GIEB) intervention on students’ posttest physics achievement while controlling for pretest scores. As shown in Table 9, the ANCOVA results indicated a significant difference between the two groups on adjusted posttest scores. The experimental group (GIEB) obtained a mean score of 48.133, which was significantly higher than that of the traditional lecture (TL) group (M = 42.033), *F*(1, 57) = 29.165, *p* < 0.001, η^2^ = 0.338.

**Table 9:** Descriptive data and ANCOVA results for the physics achievement posttest.

Variable	Group	*N*	Mean	S.D	Adjusted mean	*F*	*P*	η^2^
Post-test	GIEB	30	48.133	6.049	48.227	29.165	<0.001	0.338
	TL	30	42.033	6.178	41.940			

The effect size (η^2^ = 0.338) reflects a large effect, demonstrating that the GIEB intervention was markedly more effective than the traditional lecture method in enhancing students’ physics achievement.

## 5 Discussion

This study compared two contrasting modes of implementing metacognitive self-regulation strategies (MSRS) in Chinese junior high school physics education: a technology-enhanced mode delivered through a Gamified Interactive E-Book (GIEB) that integrates structured metacognitive scaffolding with gamification features, versus a conventional classroom mode in which the same strategies were delivered through oral teacher instruction. Using a quasi-experimental pretest–posttest design, the results indicated that students in the GIEB group demonstrated significantly greater improvements across all three outcome measures—physics achievement, metacognitive self-regulation ability, and learning motivation—than those in the TL group. Although students in the TL group showed modest gains, these changes did not reach statistical significance. These findings can be further interpreted through cyclical self-regulated learning models (e.g., Zimmerman, 2000; Pintrich, 2004), which emphasize iterative interactions among planning, monitoring, and self-reflection.

From an instructional design perspective, these findings highlight the pedagogical potential of technology-enhanced MSRS implementation. Importantly, the observed advantages of the GIEB should be interpreted as reflecting the combined effect of an integrated instructional package—comprising structured metacognitive scaffolding, gamification elements, immediate feedback, and digital visualization—rather than the isolated contribution of any single design component. The results suggest that when metacognitive strategies are delivered through an environment that simultaneously supports motivational engagement and regulatory skill development, the conditions for effective MSRS implementation are substantially improved compared to conventional oral instruction.

It is important to acknowledge that the observed improvements in the GIEB group may have been partly influenced by novelty effects. As the GIEB represented a new and unfamiliar learning format for participants, initial curiosity and excitement associated with the digital gamified environment may have contributed to heightened engagement and more favorable self-reported motivation, particularly during the early stages of the intervention. Prior research has noted that technology-based educational interventions often produce inflated short-term effects that diminish as learners become habituated to the novel medium (Clark, 1983; Sailer and Homner, 2020). Given the relatively short duration of the present intervention (4 weeks), it is not possible to determine the extent to which the observed gains reflect enduring instructional effects versus transient novelty-driven engagement. This consideration warrants caution in interpreting the magnitude of the reported effects and underscores the need for longer-duration studies that track learning outcomes beyond the initial novelty period.

A noteworthy pattern across the three outcome measures deserves attention. All three dependent variables showed statistically significant improvements in the GIEB group, with effect sizes ranging from η^2^ = 0.259 (metacognitive self-regulation) to η^2^ = 0.338 (physics achievement). The fact that physics achievement yielded the largest effect size while metacognitive self-regulation yielded the smallest is theoretically interpretable: behavioral and performance-level outcomes (i.e., test scores) may respond more rapidly to structured instructional interventions, whereas deep cognitive-regulatory capacities such as metacognitive self-regulation typically require longer periods of sustained practice and internalization to develop fully (Veenman et al., 2006; Dignath et al., 2008). Learning motivation (η^2^ = 0.286) fell between the two, consistent with its role as a mediating construct that both responds to environmental design features and supports cognitive regulation (Mega et al., 2014).

The simultaneous significance across all three measures may reflect a shared underlying mechanism. Drawing on Mega et al. (2014), who demonstrated recursive relationships among motivation, self-regulation, and academic performance, the GIEB’s design may have activated a positive feedback loop: gamification features enhanced motivational engagement, which in turn supported more active deployment of metacognitive strategies, ultimately leading to improved physics achievement. This interpretation aligns with the theoretical rationale presented in the Introduction, whereby gamification was conceptualized as motivational infrastructure for sustaining metacognitive self-regulation. Equally notable is the absence of significant gains in the TL group across all three measures. This finding suggests that oral metacognitive strategy instruction alone—without structured scaffolding, immediate feedback, or progress visualization—may be insufficient to activate the motivational and regulatory processes needed for measurable improvement, particularly within a four-week intervention window. This interpretation is consistent with prior research indicating that abstract or decontextualized strategy instruction often fails to translate into observable learning gains (Education Endowment Foundation, 2020).

It should also be noted that separate ANCOVAs were conducted for each of the three dependent variables without applying corrections for multiple comparisons (e.g., Bonferroni adjustment). This decision was based on the rationale that the three outcome measures—physics achievement, learning motivation, and metacognitive self-regulation ability—represent theoretically distinct constructs rather than repeated measures of a single construct, and each was tested against a separate, pre-specified hypothesis (Perneger, 1998). Nevertheless, conducting three independent tests at *α* = 0.05 increases the familywise error rate, and readers should interpret the significance of individual comparisons with this consideration in mind. Furthermore, the observed effect sizes (η^2^ = 0.259 to 0.338) should be interpreted with caution given the relatively small sample size (*N* = 60). Small samples tend to produce upwardly biased estimates of η^2^ (Levine and Hullett, 2002), and the true population effect sizes may be smaller than those reported here. Additionally, the absence of statistically significant gains in the TL group should not be interpreted as definitive evidence that traditional instruction produced no learning effects; given the limited statistical power associated with the present sample size—particularly for detecting small-to-medium effects—it is possible that meaningful but modest improvements went undetected.

### 5.1 Learning motivation

The findings indicate that the GIEB was associated with significant improvements in students’ learning motivation, which is consistent with prior research combining metacognitive strategy instruction with gamified learning designs (Chen C. et al., 2024; Li et al., 2022). Rather than suggesting that gamification elements directly generated intrinsic motivation, the motivational gains observed in this study may be explained by the autonomy-supportive and competence-signaling features embedded in the GIEB design, which are consistent with the principles of Self-Determination Theory (SDT).

In particular, features such as task choice, real-time feedback, and visualized progress tracking may have enhanced students’ perceptions of control and task involvement during learning activities. Previous studies have shown that when learners experience greater agency and receive informative feedback about their progress, they are more likely to sustain engagement and internalize learning goals (Karaoğlan Yılmaz et al., 2018; Mohammadi et al., 2022; Susantini et al., 2021). From this perspective, the motivational effects observed in the GIEB group can be interpreted as emerging from design conditions that supported motivational regulation rather than from reward mechanisms alone.

In contrast, the TL approach, characterized by teacher-centered instruction and limited opportunities for learner control or immediate feedback, resulted in comparatively smaller and non-significant motivational gains. This pattern aligns with previous findings indicating that instructional environments lacking explicit support for planning, monitoring, and regulation often fail to sustain students’ motivation over time (Dekker et al., 2016; Dabbagh and Kitsantas, 2013).

Viewed through the lens of Zimmerman’s cyclical model of self-regulated learning, the motivational benefits of the GIEB may reflect enhanced learner engagement across the forethought and performance phases, where goal setting, task choice, and feedback play a critical role in sustaining motivational momentum. These findings also lend support to the theoretical proposition advanced in this study: that gamification can serve as motivational infrastructure for metacognitive self-regulation. The TL group’s non-significant motivational gains suggest that oral strategy instruction, in the absence of autonomy-supportive design features and visible progress indicators, may be insufficient to counteract the motivational decline commonly observed during adolescence (Katsantonis, 2024). Future research may further examine how individual learner characteristics, such as prior motivation or cognitive style, moderate responsiveness to specific design features of gamified interactive e-books.

### 5.2 Metacognitive self-regulation ability

The GIEB intervention also resulted in significant improvements in students’ metacognitive self-regulation ability, corroborating prior studies that highlight the effectiveness of integrating metacognitive scaffolding with gamified learning environments (Al-Hosni et al., 2024; Chen Y. C. et al., 2024; Chen C. et al., 2024). These findings suggest that the structured design of the GIEB provided conditions conducive to the development and application of regulatory strategies.

From the perspective of Tang and Kay’s (2014) Metacognitive Scaffolding Gamification Framework, the GIEB embedded explicit supports for planning, monitoring, and reflection throughout the learning process. Strategy prompts, real-time feedback, and reflective activities may have helped students externalize regulatory processes and gradually internalize effective learning strategies. Rather than confirming specific causal mechanisms, the present results suggest that such scaffolded environments can facilitate the enactment of metacognitive regulation during learning tasks.

Landers (2014) mediating model of gamification provides an additional interpretive lens, proposing that gamification influences learning outcomes indirectly through mediators such as engagement, feedback processing, and strategy use. In the context of this study, gamification elements likely functioned as contextual supports that made regulatory behaviors more visible and actionable, thereby supporting students’ strategic engagement with physics content.

In contrast, the TL condition offered limited opportunities for explicit strategy guidance, timely feedback, or learner-controlled regulation, which may explain the absence of significant gains in metacognitive self-regulation. Consistent with previous critiques of conventional strategy instruction, metacognitive development appears less likely to occur in environments where regulatory processes remain implicit and unsupported (Eggers et al., 2025; Dekker et al., 2016).

Within Zimmerman’s cyclical SRL framework, the observed gains suggest that the GIEB may have supported learners’ engagement across the performance and self-reflection phases by promoting monitoring, feedback interpretation, and adaptive strategy adjustment. Notably, the effect size for metacognitive self-regulation (η^2^ = 0.259) was the smallest among the three outcome measures. This pattern is consistent with the view that deep regulatory capacities develop gradually through sustained practice, and a four-week intervention may capture only the initial stages of regulatory skill acquisition (Veenman et al., 2006). That significant improvement was nevertheless observed within this timeframe suggests that the GIEB’s structured scaffolding—particularly the visualization of learning trajectories and the prediction–verification–reflection cycle—may have accelerated the externalization and initial internalization of regulatory processes. By contrast, the TL group’s non-significant gains in metacognitive self-regulation are consistent with the implementation bottleneck identified in this study: oral strategy instruction without immediate feedback, progress visualization, or structured prompts provides insufficient support for learners to translate metacognitive knowledge into active regulatory behavior. Future studies employing larger samples, longer intervention durations, and mediation analyses could further clarify the pathways through which gamified metacognitive scaffolds influence regulatory development.

### 5.3 Physics achievement

The results further demonstrated that students in the GIEB group achieved significantly higher gains in physics achievement than those in the TL group, extending prior evidence on the academic benefits of integrating metacognitive strategies with gamified learning designs (Ateş and Polat, 2025; Chen Y. C. et al., 2024; Chen C. et al., 2024). These achievement gains may be interpreted through a combination of cognitive, behavioral, and motivational processes.

At the cognitive level, the GIEB provided a structured “planning–execution–reflection” learning pathway that may have supported students’ strategic processing and conceptual understanding during physics problem solving. At the behavioral level, visual dashboards and adaptive task pathways likely encouraged students to monitor progress and align learning behaviors with instructional goals. At the motivational level, challenge-based tasks and immediate feedback may have supported sustained task engagement.

According to Landers (2014) mediating model, gamification elements do not directly cause learning gains but may influence achievement by shaping mediating variables such as engagement, feedback use, and strategy deployment. In this study, the design features of the GIEB may have activated such mediating processes, thereby supporting students’ physics learning. Importantly, these interpretations remain tentative, as the present study did not directly test mediation pathways.

By contrast, the TL group exhibited only modest achievement gains, consistent with prior research indicating that traditional instruction often lacks the structured regulatory support and feedback necessary to translate strategy instruction into measurable academic outcomes (Schraw et al., 2006; Dekker et al., 2016).

From the perspective of Zimmerman’s SRL model, the achievement advantages associated with the GIEB suggest enhanced coordination across the forethought, performance, and self-reflection phases, enabling students to plan effectively, monitor understanding, and adjust strategies during learning. The largest effect size among the three outcome measures (η^2^ = 0.338) was observed for physics achievement, which may reflect the cumulative impact of both motivational enhancement and regulatory skill development on task performance. This finding is consistent with the theoretical proposition that gamification functions as motivational infrastructure: by sustaining engagement and making regulatory processes actionable through structured scaffolding, the GIEB may have facilitated a more complete translation of metacognitive strategies into measurable academic gains. In contrast, the TL group’s marginal achievement gains reinforce prior findings that traditional instruction often lacks the structured feedback and regulatory support necessary to convert strategy instruction into performance improvement (Schraw et al., 2006; Dekker et al., 2016). These findings collectively support the instructional value of integrating gamified design features with metacognitive self-regulation scaffolds in junior high school physics education.

### 5.4 Pedagogical implications

The findings of this study carry several practical implications for teachers, school administrators, and policymakers seeking to integrate gamified metacognitive interventions into science education.

For classroom teachers, the GIEB model demonstrates that embedding metacognitive scaffolding within a gamified digital environment can be more effective than oral strategy instruction alone. Teachers adopting similar approaches should focus on three design priorities: (a) making metacognitive processes visible through structured prompts and dashboards rather than relying solely on verbal reminders; (b) providing immediate, diagnostic feedback that helps students identify specific misconceptions and adjust strategies in real time; and (c) incorporating autonomy-supportive features—such as learning path choices and no-penalty retry mechanisms—that sustain student engagement without imposing controlling incentive structures.

For school administrators and curriculum planners, the study highlights the feasibility of implementing gamified interactive e-books within existing curriculum frameworks. The GIEB was aligned with China’s national physics curriculum standards, suggesting that gamified metacognitive tools can complement rather than replace standard instructional materials. Successful implementation, however, requires adequate technological infrastructure (e.g., tablet or computer access for all students), teacher training on facilitating technology-enhanced instruction, and ongoing technical support to ensure platform stability during classroom use.

For policymakers, the results underscore the potential of gamification-based metacognitive interventions to address persistent challenges in STEM education, including declining student motivation during adolescence and the inconsistent effectiveness of traditional strategy instruction. Policy initiatives aimed at promoting digital transformation in education could benefit from incorporating evidence-based gamification design principles—grounded in self-determination theory and self-regulated learning frameworks—into guidelines for educational technology development and procurement. Furthermore, pilot programs that evaluate gamified metacognitive tools across diverse school contexts (urban vs. rural, well-resourced vs. under-resourced) would help identify scalability challenges and inform context-sensitive implementation strategies.

It should be noted that the sustainability of gamified interventions depends on ongoing content updates, alignment with evolving curriculum standards, and continuous monitoring of student engagement patterns to prevent habituation effects. Long-term implementation plans should therefore include mechanisms for iterative refinement based on formative evaluation data.

## 6 Limitations and recommendations

Although this study provides preliminary empirical evidence supporting the effectiveness of the Gamified Interactive E-Book (GIEB) incorporating Metacognitive Self-Regulation Strategies (MSRS) in improving Chinese junior high school students’ physics achievement, metacognitive self-regulation ability, and learning motivation, several limitations should be acknowledged. These limitations highlight directions for refinement and expansion in future research.

First, the study was conducted in a single public junior high school in Hefei, Anhui Province, with a relatively small sample size of 60 students. The homogeneity of the sample in terms of geographic region, school type, and socioeconomic context may limit the external validity and generalizability of the findings. Students’ acceptance of and responses to the GIEB may vary across different regions, school types, or learner backgrounds. To enhance external validity, future research should adopt several concrete strategies: (a) recruiting participants from multiple schools across different regions (e.g., eastern coastal vs. western inland provinces in China) to capture geographic and socioeconomic variability; (b) including both urban and rural school settings, as students’ prior exposure to digital learning tools and gamified environments may differ substantially; (c) expanding the sample to include students from different grade levels (e.g., Grades 7 and 9) to examine developmental differences in responsiveness to gamified metacognitive interventions; and (d) employing multi-site randomized controlled trials with larger sample sizes to provide more robust evidence of treatment effects and to enable subgroup analyses across demographic variables. Furthermore, the relatively small sample size limits statistical power and may result in inflated effect size estimates. Partial eta squared values derived from small samples tend to overestimate population effect sizes (Levine and Hullett, 2002), and the large effect sizes reported in this study (η^2^ = 0.259 to 0.338) should therefore be interpreted as preliminary estimates subject to replication with larger samples.

Second, the intervention lasted 4 weeks and primarily examined short-term learning outcomes. Although the experimental group demonstrated significant improvements in learning motivation, metacognitive self-regulation, and physics achievement, it remains unclear whether these effects can be sustained over time or transferred to other learning contexts. Future studies are encouraged to incorporate delayed posttests, longitudinal designs, or cross-disciplinary implementations to examine the durability and transferability of the observed effects.

Third, while baseline differences in learning motivation, physics achievement, and metacognitive self-regulation ability were controlled using pretests and ANCOVA, several individual learner characteristics were not included in the analytical model. Variables such as digital literacy, cognitive style, gender, and motivational orientation may moderate the effectiveness of gamified and strategy-based learning environments. Future research should consider incorporating these variables at the design stage or applying advanced analytical approaches (e.g., multilevel modeling or subgroup analysis) to improve explanatory precision.

Fourth, the physics achievement test used in this study was developed by the researchers based on national curriculum standards. Although the test was reviewed by subject-matter experts and demonstrated satisfactory reliability and content validity, it was not benchmarked against standardized assessments. This may limit the comparability of findings across studies. Future research could employ standardized or externally validated instruments to strengthen measurement robustness and cross-study comparability.

Fifth, although the GIEB was designed to align metacognitive scaffolding with SDT-informed design principles and commonly used gamification mechanisms (e.g., points, feedback, task challenges, and adaptive pathways), the present study did not disentangle the independent or interactive effects of specific design components. The GIEB and TL conditions differed simultaneously across multiple dimensions, including digital medium, structured scaffolding, immediate automated feedback, and gamification elements. Consequently, the observed effects should be attributed to the integrated instructional package rather than to any single component such as gamification alone. This design choice reflects a deliberate first-stage research strategy: as Clark (1983) and Sailer and Homner (2020) have noted, establishing the effectiveness of an integrated intervention is a necessary precondition before component-isolation studies become warranted. Future research should employ factorial or dismantling designs—for example, comparing a GIEB group, a non-gamified interactive e-book group, and a traditional instruction group—to isolate the independent contributions of gamification, digital scaffolding, and immediate feedback to the observed learning gains.

Sixth, despite efforts to standardize instructional procedures, potential confounding factors such as novelty effects and individual differences in digital competence could not be fully excluded. The GIEB introduced a new and unfamiliar learning format for participants, and the novelty of the gamified digital environment may have amplified students’ initial engagement, self-reported motivation, and task performance, particularly during the early stages of the intervention. Because the intervention lasted only 4 weeks, it was not possible to determine whether the observed effects would persist after the initial novelty diminished. Future studies should incorporate delayed posttests administered after extended periods (e.g., 2–3 months post-intervention) and include measures of platform familiarity and technology attitudes as covariates to disentangle genuine instructional effects from transient novelty-driven engagement. Additionally, pre-measures of digital skills and technology experience should be considered, or subgroup analyses conducted, to better control for technology-related variables and strengthen internal validity (Clark, 1983; Sailer and Homner, 2020).

In conclusion, this study provides preliminary theoretical and empirical support for the feasibility of integrating gamification with metacognitive self-regulation strategies in junior high school physics education. To enhance both theoretical rigor and practical sustainability, future research should refine this instructional model across multiple dimensions, including sample diversity, intervention duration, learner characteristics, assessment instruments, and gamification mechanics. Moreover, examining the adaptability of this approach across different subjects, grade levels, and learner profiles will be essential for advancing gamification-based, metacognitively supported instructional design in science education.

## 7 Conclusion

This study investigated whether gamification can serve as motivational infrastructure for sustaining metacognitive self-regulation strategies in junior high school physics education. Using a quasi-experimental design with 60 eighth-grade students in China, the effects of a Gamified Interactive E-Book (GIEB) were compared with traditional lecture-based instruction incorporating oral metacognitive strategy guidance. The GIEB group demonstrated significantly greater improvements across all three outcome measures—physics achievement (η^2^ = 0.338), learning motivation (η^2^ = 0.286), and metacognitive self-regulation ability (η^2^ = 0.259)—while the TL group showed no significant gains on any measure.

These findings provide empirical support for the central proposition of this study: that the sustained implementation of metacognitive self-regulation strategies requires continuous motivational support and immediate feedback mechanisms that traditional instruction struggles to provide. The GIEB’s structured “planning–monitoring–reflection” scaffold, combined with autonomy-supportive gamification features and visualized progress tracking, appears to have activated a positive feedback loop in which motivational engagement supported more active deployment of metacognitive strategies, which in turn facilitated improved physics achievement. By contrast, the TL group’s null results suggest that oral strategy instruction alone is insufficient to overcome the motivational bottleneck that limits MSRS effectiveness in conventional classrooms.

From a theoretical perspective, this study contributes to the literature by reframing gamification not merely as a motivational add-on but as a design scaffold that supports learners’ engagement across the full cycle of self-regulated learning—forethought, performance, and self-reflection (Zimmerman, 2000). The differential pattern of effect sizes across the three outcome measures further suggests that behavioral outcomes respond more rapidly to structured interventions, whereas deep regulatory capacities require longer periods of sustained practice to develop fully.

Several limitations qualify these findings, including the small sample size, short intervention duration, single-school context, and the inability to disentangle the independent contributions of specific gamification elements. These constraints, detailed in the Limitations section, underscore the need for larger-scale, longer-duration studies employing factorial designs and mediation analyses to clarify the mechanisms through which gamified metacognitive scaffolds influence learning outcomes.

Future research should examine the applicability of the GIEB model across diverse STEM subjects, school contexts, and learner populations, with particular attention to the durability of observed effects through delayed posttests and longitudinal designs. Investigating moderating variables such as prior motivation, digital literacy, and cognitive style would further refine understanding of for whom and under what conditions gamified metacognitive interventions are most effective. Such efforts will contribute to the development of scalable, theory-grounded instructional models that integrate gamification with metacognitive self-regulation support in digital science education.

## Data Availability

The raw data supporting the conclusions of this article will be made available by the authors, without undue reservation.
